# An Analysis of G3BP2 in Non-Small Cell Lung Cancer

**DOI:** 10.3390/cancers18060969

**Published:** 2026-03-17

**Authors:** Leela S. S. Bandi, Leah Timon, Elena O’Toole, Diarmuid O’Connor, Kristen Andersen, Bashir M. Mohamed, Siobhan Nicholson, Gerard J. Fitzmaurice, Ronan Ryan, Vincent Young, Sinead Cuffe, Stephen P. Finn, Steven G. Gray

**Affiliations:** 1 Thoracic Oncology Research Group, Central Pathology Laboratory, St. James’s Hospital, D08 W9RT Dublin, Ireland; 2 Department of Histopathology, Labmed Directorate, St. James’s Hospital, D08 RX0X Dublin, Irelandbmohamed@tcd.ie (B.M.M.);; 3 Surgery, Anaesthesia and Critical Care Directorate, St James’s Hospital, D08 NHY1 Dublin, Ireland; 4 HOPE Directorate, St James’s Hospital, D08 NHY1 Dublin, Ireland; 5 Department of Histopathology and Morbid Anatomy, Trinity College Dublin, D08 RX0X Dublin, Ireland; 6 Department of Clinical Medicine, Trinity College Dublin, D08 W9RT Dublin, Ireland

**Keywords:** G3BP2, stress granule, non-small cell lung cancer

## Abstract

Background/Objectives: Cancer cells are subjected to various stress conditions and have stress adaptability strategies to survive. Various types of stresses lead to the aggregation of cytoplasmic RNA granules known as stress granules (SGs), seen in normal and tumor cells, and aid in cell survival by avoiding cell apoptosis. G3BP stress granule assembly factor 2 (G3BP2) encodes a multifunctional protein with known roles as a critical component of SGs and is also associated with chemoresistance in cancer, but its known roles in non-small cell cancer (NSCLC) are limited. Methods: We evaluated the expression of G3BP2 via qPCR and immunohistochemistry on a retrospective cohort of NSCLC isolated at surgery in St James’s Hospital, Dublin, Ireland. Expression levels were correlated with clinicopathological parameters. Survival analyses, including Kaplan–Meier analyses, were used to determine the prognostic value. Additional correlations with other available NSCLC datasets were explored. Results: In contrast to other studies, we did not observe upregulated expression of G3BP2. Furthermore, Kaplan–Meier analyses did not identify any prognostic value associated with G3BP2 expression in patient tissues in contrast to other published data. Bioinformatic analyses on these other datasets found strong correlations between G3BP2 and core stress granule genes in NSCLC. Additional analyses also identified correlations between G3BP2 expression and immune cell infiltration, immune cell exhaustion, and DNA Damage Response pathways. An examination of the available datasets did not find any overall prognostic value for altered DNA methylation and survival. However, two individual CpG residues were identified for which higher methylation was associated with worse overall survival. Finally, the effects of a G3BP2 inhibitor on cellular proliferation were assessed. Conclusions: In our analysis, G3BP2 was not associated with survival benefit. However, clear associations were observed between altered expression of this gene and a number of important pathways linked to cancer pathogenesis, and further studies are warranted to assess this gene (and/or) stress granules in cancer.

## Simple Summary

Exposure to stress can make cells die, and cells develop coping strategies to prevent this. One of the things that can lead to this stress in a cancer cell is an overproduction of messages (called mRNA) which overwhelms the cells’ ability to turn these messages into protein. To stop this, cells respond by pulling these mRNAs into a complex called a stress granule, which stops the cells from making these messages into protein. In cancer, this stress granule response can, however, lead to cancer cell survival and poor outcomes for cancer patients. An essential molecule found in stress granules is a protein called G3BP2. This study examines G3BP2 in lung cancer, and the results provide a better understanding of how this protein affects lung cancer and how we may be able to target this protein for patient benefit.

## 1. Introduction

Lung cancer is a major public health issue, representing 12.4% of all cancer cases diagnosed all over the world [1]. According to GLOBOCAN 2022 estimates by the International Agency for Research on Cancer (IARC), lung cancer is the second most prevalent cancer, with 2.21 million new cases, and the deadliest form of cancer, with around 1.8 million deaths (18.7%) in the year 2022 [1]. Lung cancer is loosely categorized into two primary types: non-small cell lung cancer (NSCLC), found across approximately 85% of patients, and small cell lung cancer (SCLC), accounting for the other 15% of patients. Within NSCLC, lung adenocarcinoma (LUAD) is the most common subtype of NSCLC, followed by lung squamous cell carcinoma (LUSC) [2]. Advances in the management of NSCLC have resulted in a decrease in overall mortality over the years, influenced by the adoption of lung cancer screening through low-dose CT scan and genetic mutational screening, reduced smoking rates, and significant advancements in targeted therapies and immunotherapies [3].

Cellular stress is common in cancer [4], and indeed it is most likely that cellular stress mechanisms can be linked between all of the current hallmarks of cancer [5,6,7]. Key examples of such stress conditions include oxidative stress, endoplasmic reticulum (ER) stress, heat shock and hypoxia [8].

One such stress involves aberrant messenger RNA (mRNA) translation, as tight control of messenger RNA (mRNA) processing, trafficking, degradation, and translation is critical for regulating gene expression [9]. Stress granules (SGs) form when stress-activated pathways stall translation initiation and form assemblies of non-translating messenger ribonucleoproteins (mRNPs) [10]. SGs contribute to many of the hallmarks of cancer including proliferation, invasion, migration, avoiding apoptosis, metabolism reprogramming and immune evasion [5,8]. SGs are dynamic, and their formation and dissolution reflect changes in mRNA metabolism, thus allowing cells to regulate their proteome and/or mediate life or death decisions during changing environmental conditions [9]. It is now well established that persistent or aberrant stress granule formation play important roles in carcinogenesis [8]. Evidence suggests that cancer cells use stress granules to protect mRNAs that regulate vital cellular processes from degradation [11], representing a highly conserved cellular strategy to reduce stress-related damage and promote cell survival. This mechanism promotes tumor cell survival, proliferation, invasion, migration, and allows for evasion of apoptosis, leading overall to tumor progression. Additionally, stress granules contribute to resistance against chemotherapy and radiotherapy, reducing treatment effectiveness, while simultaneously regulating the tumor immune microenvironment, aiding in immune evasion. Thus, stress granules play crucial roles in tumor development and response to therapy [8,12].

A central element in the makeup of SGs is a large number of RNA-binding proteins (RBPs) [10]. From a recent detailed proteomic analysis of SGs, it has emerged that SG formation is encoded by a core protein–RNA interaction network [13]. From this analysis, it emerged that two key RBPs, GTPase-activating protein-binding protein 1 (G3BP1) and GTPase-activating protein-binding protein 2 (G3BP2), have the highest centrality within this network and are essential for SG assembly under certain conditions [13]. Both G3BP1 and G3BP2 have both been linked to the pathogenesis of NSCLC [14,15,16,17,18,19].

ER stress is another link to SG formation [20]. We recently demonstrated that the expression of USO1 a protein associated with ER–Golgi trafficking is both dysregulated and prognostic in NSCLC [21]. The cytogenetic location of both USO1 and G3BP2 is Chromosome 4q21. Given the potential links between USO1, the ER, ER stress and SGs, we set out to assess the expression of G3BP2 in NSCLC to determine if its expression also has any potential utility as a biomarker in lung cancer at both the mRNA and protein levels. Our analysis also examined links between *G3BP2* mRNA expression and other oncogenic driver genes commonly associated with NSCLC, along with the identification of novel mutated genes that affect *G3BP2* mRNA expression. Correlations between G3BP2 mRNA expression and various parameters such as tumor mutational burden and immune cell infiltration were explored, while links between CpG methylation at individual CpG residues of the G3BP2 gene and overall survival (OS) were identified. Finally, we assessed if a known G3BP2 targeting agent (Compound C108) [22] could be potentially used to target NSCLC.

## 2. Materials and Methods

### 2.1. Nomenclature

Nomenclature for genes and proteins are presented according to the current HUGO definitions [23].

### 2.2. Cell Culture

Eight cell lines containing a number of normal human bronchial epithelial cell lines (HBECs) and NSCLC cell lines were used in this study. This panel comprised normal bronchial epithelial cells (HBEC3, HBEC4 and HBEC5) and NSCLC cell lines as follows: NCI-H1975, and NCI-H1819 (adenocarcinoma). Another panel of isogenic parent cells resistant to cisplatin comprised the following: A549 (adenocarcinoma), SKMES1 (squamous cell carcinoma) and DLKP (squamous cell carcinoma).

Routine Growth of cells were maintained in a humidified atmosphere containing 5% CO_2_ in appropriate media supplemented with 10% fetal bovine serum (FBS) and Antibiotic Antimycotic Solution (Sigma-Aldrich, St. Louis, MO, USA: Cat No: A5955), with the exception of HBECs. HBECs were grown on collagen-coated plates using the Keratinocyte serum free media (K-SFM (1X) Kit (Gibco™; Cat. No: 17005042, Waltham, MA, USA). A549 cells were cultivated in Nutrient Mixture F-12 Ham (Sigma-Aldrich, St. Louis, MO, USA—Cat. No: N6658). SKMES-1 cells were maintained in Dulbecco’s Modified Eagle’s Medium (DMEM)—high glucose (Sigma-Aldrich, St. Louis, MO, USA—Cat. No: D6429). DLKP cells were grown in DMEM/Ham’s F12 (1:1) (Sigma-Aldrich, St. Louis, MO, USA: Cat No: D8437. All other NSCLC cell lines were maintained in RPMI-1640 Medium (Sigma-Aldrich, St. Louis, MO, USA—Cat. No: R8758). All cell lines were routinely tested for mycoplasma as per the published PCR protocol [24].

### 2.3. Primary Tumor Samples

This study utilized a number of surgically resected chemotherapy-naïve tumor specimens. At resection, all samples were evaluated by a pathologist, and tumor tissue samples along with associated matched normal tissue were isolated and flash-frozen for downstream analysis. Informed consent for bio-banking was obtained from each patient prior to surgery, and for retrospective analyses individual consent was waived. This study proceeded only after formal approval from the SJH/AMNCH Hospital Ethics Committee—Ethics REC (No.: 041018/8804) and in accordance with the Declaration of Helsinki (as revised in 2013). Twenty matched normal/tumor specimens were examined, comprising 10 adenocarcinomas and 10 squamous cell carcinomas, and a summary of their clinical and histopathological data is summarized in Table 1.

### 2.4. Formalin Fixed Paraffin Embedded Samples

A tissue microarray (TMA) with a total of 204 surgically resected NSCLC tumor specimens from the period 1999–2007 (Ethics REC No.: 041018/8804) was utilized in this study. Surgically resected tumor specimens and control specimens were fixed with 10% formalin and embedded in paraffin (FFPE). Samples were staged using The Union for International Cancer Control Tumor-Node-Metastasis (TNM) Classification of Malignant Tumors 8th edition [25,26] and subsequently subtyped histologically using World Health Organization (WHO) guidelines [27]. The available clinical and histopathological data (including age, sex, smoking status, histology, TNM stage, surgical procedure, tumor grade, and primary site) for the patients used in this TMA have previously been described by us [21,28,29]. The TMA itself was generated using a Beecher Manual Tissue Arrayer, Model MTA-1 (Estigen OÜ, Tartu, Estonia), and for each patient quadruplicate cores (0.6 mm) of FFPE sample were embedded and 4 µm sections were sectioned and stained for immunohistochemistry (IHC) analysis of G3BP2.

### 2.5. Immunohistochemistry

IHC was performed on TMA sections by utilizing a standard protocol to deparaffinize, rehydrate and wash the slides. Subsequently, ULTRA cell conditioning (ULTRA CC1), pH 9.1, was applied for 92 min at 100 °C for heat-induced epitope retrieval (HIER). For G3BP2 antibody staining, rabbit polyclonal primary antibody G3BP2 (PA5-53776, Invitrogen, ThermoFisher Scientific, Waltham, MA, USA), was used. The primary antibody was diluted in Roche antibody diluent (Catalog #: 05261899001 (Roche Diagnostics, Basel, Switzerland)) (1:250) and was applied to the sections for 32 min at ambient temperature and stained using the OptiViewTM DAB IHC detection kit (Roche Diagnostics, Basel, Switzerland, Cat# 06396500001), in conjunction with the Optiview Amplification Kit (Roche Diagnostics, Basel, Switzerland, Cat# 06396518001) on a Ventana BenchMark ULTRA immunohistochemistry staining system (Roche Diagnostics, Basel, Switzerland, Cat# 05342716001).

Following IHC, a staining intensity score was determined by two pathologists blinded to the clinical, pathological and follow-up data. Staining intensity was designated as either 0, 1+, 2+ or 3+, and each tumor section was given an H score between 0 and 300 = 3(% at 3+) + 2(% at 2+) + 1(% at 1+). Staining intensity was then scored for all available cores, ranging from 0 to 285, and no samples reached the maximum overall H Score of 300.

High G3BP2 expression was designated as those with an average H score equal to or above the median value, and low expression was designated as those H scores below the median. To construct survival curves, Kaplan–Meier analyses were conducted using Prism 5.01 (GraphPad, San Diego, CA, USA).

### 2.6. Isolation of RNA, RT-PCR and qPCR Analyses

Total RNA was isolated and converted to complementary DNA (cDNA) using protocols and methodology as described previously [29]. Briefly, total RNA was extracted using TRI reagent (Molecular Research Center, Montgomery Road, OH, USA). To generate cDNA, 250 ng of total RNA was then pre-treated with amplification grade DNase I (Sigma-Aldrich, St. Louis, MO, USA—AMPD1-1KT) in order to remove contaminating genomic DNA, and subsequently, a ReadyScript^®^ cDNA Synthesis Mix (Sigma-Aldrich, St. Louis, MO, USA—Cat RDRT) was used to generate the first-strand cDNA according to the manufacturer’s instructions. All procedures followed the manufacturer’s instructions.

G3BP2 expression was quantified by real-time qPCR (absolute quantification method) on a Prime Pro 48 qPCR system (Bibby Scientific Ltd. Beacon Road, Stone, Staffordshire, UK) and 2× ChamQ Universal SYBR qPCR Master Mix (Vazyme, Red Maple Hi-tech Industry Park, Nanjing, China, Cat#Q711-03) using the manufacturer’s protocol in a two-step qPCR program with a synthesized GBlock™ (IDT Integrated DNA Technologies, Inc., Coralville, IA, USA) for G3BP2 as the standard using the following primers:

G3BP2 FWD: 5′-TCCTCCTAGAGGACCAAGAC-3′

G3BP2 REV: 5′-GATGACTATCTGGATAGCGAAT-3′

Cycling parameters were as follows: Polymerase activation at 95 °C for 2 min followed by 35 cycles of 95 °C for 15 s and annealing/amplification at 55 °C for 45 s. Outputs were analyzed using the default in-built StepOne software (Version 2.3), exported, and graphed using Prism 5.01.

Endpoint RT-PCR to examine G3BP2 was also conducted using the same primers and amplification conditions in a Kyratec Supercycler (Mansfield, Queensland, Australia).

18S rRNA was used as a housekeeping gene/loading control and amplified using the following primers as per [30].

18S rRNA Forward 5′-GATGGGCGGCGGAAAATAG-3′

18S rRNA Reverse 5′-GGCGTGGATTCTGCATAATGG-3′

The same amplification protocol was used for RT-PCR as above but with the annealing temperature increased to 58 °C and the number of cycles reduced to 30. All PCR products were gel electrophoresed on a 3% agarose gel, and the PCR products were visualized using an LI-COR ODYSSEY FC imaging system (LI-COR, Lincoln, NE, USA).

### 2.7. In Silico Validation of Expression Differences for G3BP2 in NSCLC

Assessment of G3BP2 mRNA and protein expression was examined using cProSite [31] to interrogate the datasets of the National Cancer Institute’s Clinical Proteomic Tumor Analysis Consortium (CPTAC), allowing for analysis of paired tumor and normal samples with associated gene expression and proteomic data such as protein phosphorylation [32,33].

### 2.8. In Silico Survival Analysis

KM-Plot was utilized to assess if G3BP2 mRNA expression had any prognostic value with respect to patient survival in available online datasets [34]. A univariate analysis was used incorporating a Cox proportional hazards model with expression separated at the median as the cut-off. Subsequently, a separate analysis of the TCGA-LUAD and TCGA-LUSC datasets for survival associated with a 7 gene-Key SG gene signature or a 36 gene “core” SG gene signature as defined by Yang et al. [13] was conducted using GEPIA3 [35]. As a caveat, the general reader should be aware that one issue arising from this analysis is the potential for conflicting results based off the datasets themselves. For instance, KM-Plot analyses utilize microarray data for one specific probe (208841_s_at), while GEPIA3 analyses utilize NGS sequencing data. As such, the probe used in KM-Plot analyses may miss different mRNA isoforms, potentially skewing outputs.

To assess if DNA CpG methylation at the G3BP2 gene was associated with altered gene expression, cBioPortal was used [36]. For analysis of whether individual CpG residues were further associated with potential survival benefit, MethSurv was utilized [37]. To determine if G3BP2 mRNA expression is associated with immunotherapy survival benefit, analysis of a pan-cancer dataset was conducted using KM-Plot as per Kovács et al. [38].

### 2.9. Associations Between Key Oncogenic Driver Mutations, Novel Mutated Genes and GBP2 Expression

An overall assessment of alterations for genetic alterations to G3BP2 was conducted using cBioPortal [36]. To further assess if any correlations exist between G3BP2 mRNA and the expression of key genes in NSCLC that are commonly associated with altered expression, or oncogenic driver mutations were examined using TIMER3 [39] with the modules Gene_Corr, and Gene_Mutation. The muTarget platform was also used to examine the datasets containing RNA-sequencing and mutation data to determine if G3BP2 expression was affected by novel mutated genes compared to the corresponding wild-type gene expression. Analysis in this instance was conducted using G3BP2 as the target gene and with mutation prevalence set at 2% [40]. Results were validated using correlation analysis on the TIMER3 portal [39].

### 2.10. GPBP2 Expression, Tumor Mutational Burden and Immune Infiltrations in NSCLC

cBioPortal was used to examine if there were any correlations between tumor mutational burden (TMB) and G3BP2 mRNA expression [36]. To further assess TMB, we utilized either a panel of proxy markers of tumor mutational burden and analysis in GEPIA3 [35].

### 2.11. Drug Assessment

To determine if pharmacological inhibition of G3BP2 had any potential for therapeutic intervention, HBECs and NSCLC cell lines were exposed to C108, a small molecule that binds to G3BP2 and interferes with stress response [41]. Compound C108 was purchased from Sigma-Aldrich (St. Louis, MO, USA Cat# SML2017) and dissolved in DMSO at a final concentration of 10 mM. Cells were plated in a 96-well plate for 24 h prior to addition of Compound C108 and incubated for a further 48 h. Cellular viability was assessed using a CCK-8 assay (Selleckchem, Houston, TX, USA Cat# B34304) according to the manufacturer’s protocol.

### 2.12. Availability of Data and Materials

The data that support the findings presented in this study are available for interrogation at the following online resources:

TIMER: https://cistrome.shinyapps.io/timer/ (Tested on 19 January 2026)

TIMER3.0: https://compbio.top/timer3/ (Tested on 27 January 2026)

GEPAI3.0: https://gepia3.bioinfoliu.com/ (Tested on 19 January 2026)

KM-PLOT: https://kmplot.com/analysis/index.php?p=home (Tested on 19 January 2026)

cBioPortal: https://www.cbioportal.org/ (Tested on 19 January 2026)

muTarget: https://www.mutarget.com/ (Tested on 19 January 2026)

cProSite: https://cprosite.ccr.cancer.gov/#/ (Tested on 19 January 2026)

Methsurv: https://biit.cs.ut.ee/methsurv/ (Tested on 28 January 2026)

OncoSplicing: http://www.oncosplicing.com/ (Tested on 10 February 2026)

## 3. Results

### 3.1. Assessment of G3BP2 in NSCLC

**Figure 1:**
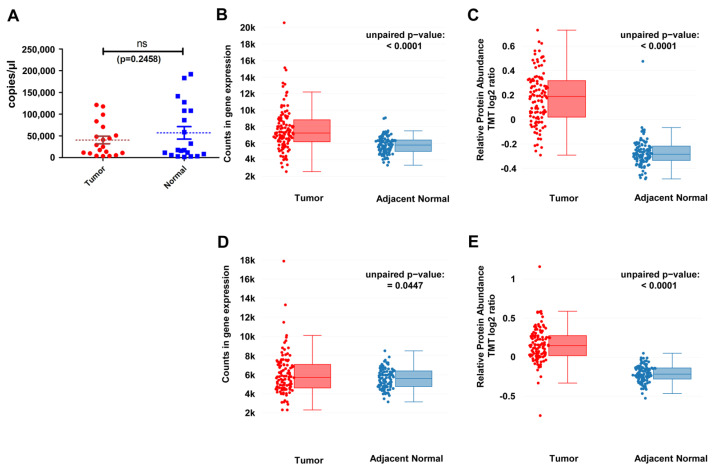
Expression of G3BP2 in NSCLC. (**A**) qPCR analysis of G3BP2 expression in a panel of matched fresh-frozen tumor/adjacent normal NSCLC samples. G3BP2 expression in LUAD in (**B**) mRNA and (**C**) protein in the CPTAC TCGA dataset; G3BP2 expression in LUSC in (**D**) mRNA and (**E**) protein in the CPTAC TCGA dataset.

We examined the expression G3BP2 mRNA in a number of surgically resected fresh-frozen normal/tumor matched patient samples (n = 20) by qPCR (Figure 1A). Overall, in our sample’s levels of G3BP2, mRNA was not significantly altered between tumor and normal (*p* = 0.2458). However, using in silico approaches we examined the expression of G3BP2 mRNA and protein in the cProSite dataset. This demonstrated that G3BP2 mRNA was elevated significantly in both LUAD (Figure 1B; *p* < 0.0001) and LUSC (Figure 1D; *p* = 0.047). The elevated expression in these samples was matched at the protein level for both LUAD (Figure 1C; *p* < 0.0001) and LUSC (Figure 1E; *p* < 0.0001). The results suggest that overall, G3BP2 levels are significantly altered in NSCLC.

Phosphoprotein analysis of the protein data in cProSite further identified several serine residues S241 and S149 in LUAD, and S149 and S253 in LUSC, which are significantly elevated in the tumors compared with their matched adjacent normal lung (Supplementary Figure S1).

### 3.2. G3BP2 Immunohistochemistry and Survival Analyses

**Figure 2:**
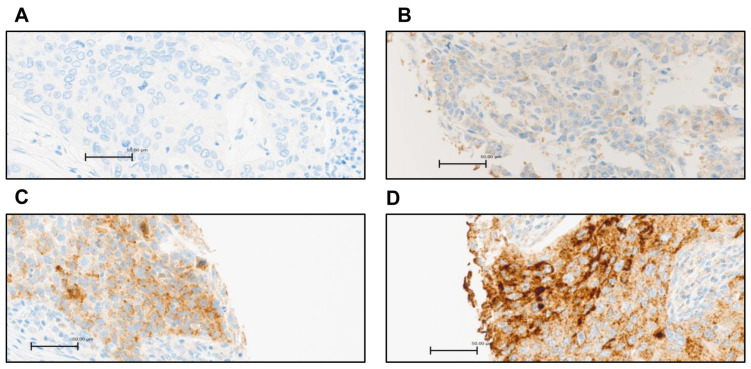
Representative examples of G3BP2 protein expression in NSCLC. (**A**) Negative staining (H score 0), (**B**) low level of expression (H score 100), (**C**) high level of expression (H score 200) and (**D**) highest level of expression (H score 300), magnification ×40; detection of G3BP2 protein by immunohistochemistry was conducted using rabbit polyclonal G3BP2 primary antibody (PA5-53776), counterstained with hematoxylin II and Bluing Reagent. Scale bar represents 50 µm.

Positive immunohistochemical staining for G3BP2 in NSCLC demonstrated increasing distribution and intensity across the H-score as shown in Figure 2. However, whilst granular cytoplasmic staining patterns are observed, it is our opinion that in order to truly ascertain if these structures are indeed SGs, a dual stain should be utilized (for example, G3BP1 and G3BP2). As such, the H-scoring used for this analysis was based solely off total intensity and distribution only.

**Figure 3:**
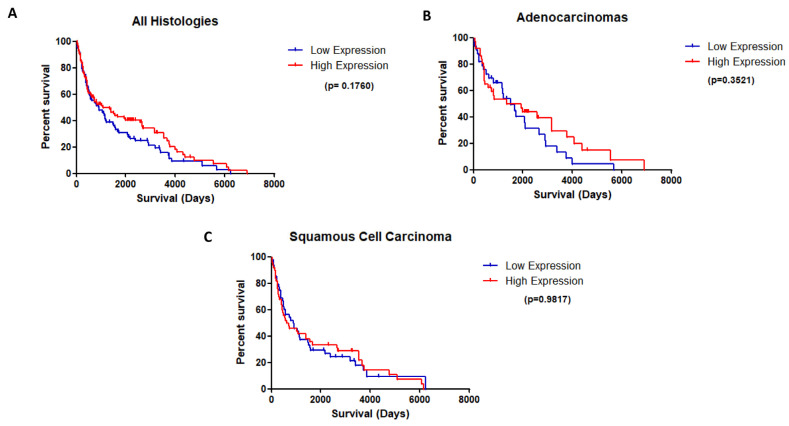
Kaplan–Meier survival analysis according to G3BP2 protein expression. Overall survival (OS) presented in (**A**) all histologies (n = 180), (**B**) adenocarcinomas (n = 70), (**C**) squamous cell carcinomas (n = 98) ((log-rank test)). Probability of survival is graphed based off H-scores ≥ 100 (red line) and <100 (blue line).

Of the available cases in the TMA staining, n = 180 patients were deemed as being appropriate for scoring. Each was given an H score, and an average score of patient cores was calculated. After all cases were scored, the median H score was calculated as 100. Tumors with scores ≥ 100 were designated as having high expression and those with scores < 100 as having low expression of G3BP2. Results were then analyzed for any overall survival benefit, and the results are presented in Figure 3.

**Figure 4:**
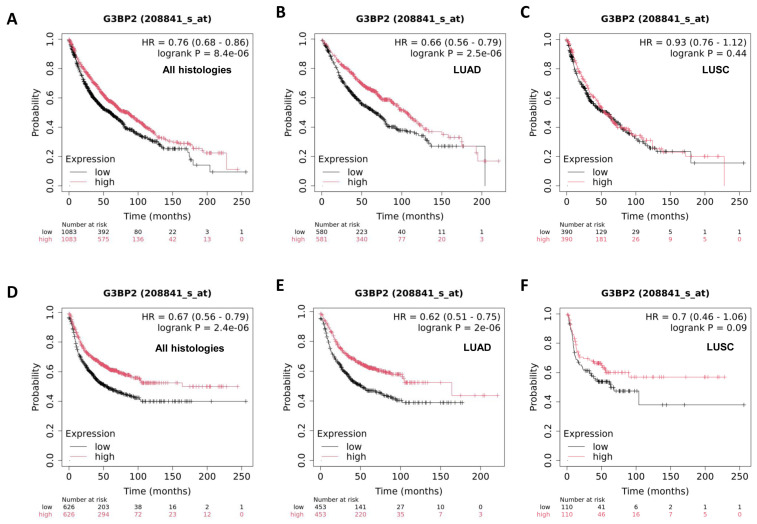
Kaplan–Meier survival analysis as derived from KM-Plot. Overall survival (OS) presented in (**A**) all histologies (n = 2166), (**B**) adenocarcinomas (n = 1161), (**C**) squamous cell carcinomas (n = 780). Progression-Free Survival (PFS) presented in (**D**) all histologies (n = 1252), (**E**) adenocarcinomas (n = 906), (**F**) squamous cell carcinomas (n = 220). PFS was generated using the available data for first progression (FP).

Overall, there was no apparent OS benefit associated with G3BP2 expression. To investigate this further, we also used KM-Plot [34] to evaluate the relationship between G3BP2 mRNA expression and both OS and PFS in LUAD and LUSC as shown in Figure 4.

The results from KM-Plot suggest that overall, across all histologies, high expression of G3BP2 mRNA is associated with both better OS (*p* = 8.4 × 10^−6^) and PFS (*p* = 2.4 × 10^−6^) (Figure 4A,D). The survival benefit, however, appears to be restricted to the lung adenocarcinoma histological subtype with better OS (*p* = 2.5 × 10^−6^) and PFS (*p* = 2 × 10^−6^) (Figure 4B,E). No apparent survival benefit could be attributed to lung squamous cell carcinoma histology (Figure 4C,F).

**Figure 5:**
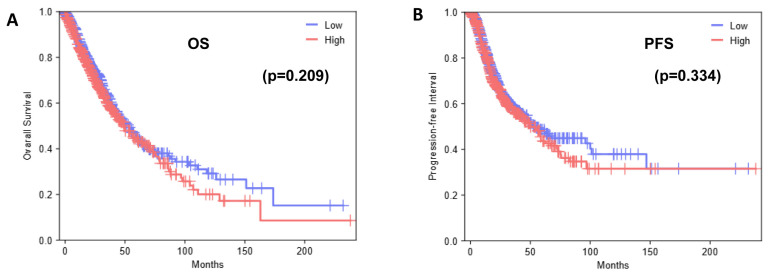
Kaplan–Meier survival analysis as derived from GEPIA3. Survival associated with G3BP2 mRNA for all histologies is presented as (**A**) OS and (**B**) PFS. Patient numbers were (n = 996) for both analyses. Analysis was conducted on GEPIA3 on 22 January 2026.

However, an analysis of GB3Bp2 mRNA expression from the TCGA datasets did not find any OS or PFS benefit as shown in Figure 5, which is more in agreement with our observations.

**Figure 6:**
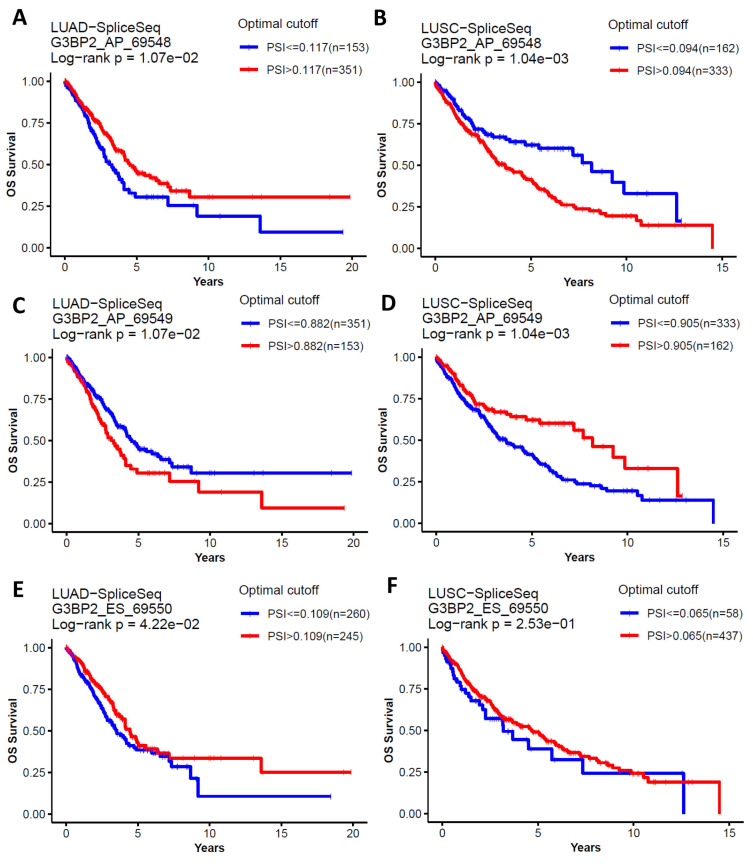
Splicing events in G3BP2 associated with OS. OncoSplicing [42] identified three splicing events associated with OS as shown. Optimal cut-off was predicted using survival data by the “surv_cutpoint” function in the R package “survminer” [42]. PSI—Percent Splice In. Alternative promoter splicing at AP_6954 is associated with better survival in (**A**) LUAD; and with worse OS in LUSC (**B**). Likewise, alternative promoter splicing at AP_69549 is associated with worse OS in LUAD (**C**), and better OS in LUSC (**D**). Finally exon skipping (ES_69550) is linked tom better OS for both LUAD (**E**) and LUSC (**F**).

To assess if G3BP2 splice variants had any prognostic value, the OncoSplicing [42] database was queried. This analysis identified three known splicing events associated with OS in LUAD and LUSC, two of which were from alternative promoters (G3BP2_AP_69548, G3BP2_AP_69549), while another was an Exon skipping event between exons 8 and exon 10 (G3BP2_ES_69550). The results are shown in Figure 6.

The first splicing event (AP_69548) is associated with better OS in LUAD (*p* = 0.0107; Figure 6A) and with worse OS in LUSC (*p* = 0.00104; Figure 6B). In contrast, the other alternative promoter splicing event (AP_69549) is associated with worse OS in LUAD (*p* = 0.0107; Figure 6C) and better OS in LUSC (*p* = 0.00104; Figure 6D). Given the survival curves and *p*-values presented, the data suggests that these two alternative promoters have inverse associations/usage in LUAD and LUSC. In contrast, the exon-skipping splice event’s (ES_69550) higher PSI was shown to be associated with better OS for both LUAD (*p* = 0.042; Figure 6E) and LUSC (*p* = 0.0253; Figure 6F).

**Figure 7:**
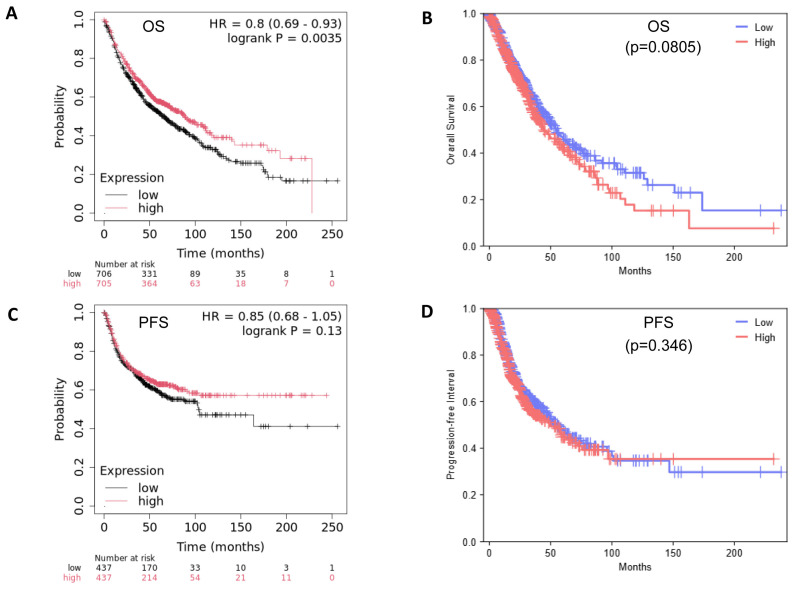
Kaplan–Meier survival analysis of a “core” 36-gene SG signature. Survival associated with G3BP2 mRNA for all histologies was assessed using KM-Plot or GEPIA3. The results are presented for KM-Plot as (**A**) OS and (**C**) PFS. Patient numbers were (n = 1411) for OS and (n = 874) for PFS analyses. The results of this gene signature as analyzed using GEPIA3 are presented as (**B**) OS and (**D**) PFS. Patient numbers were (n = 996) for both analyses. Analysis was conducted on both KM-Plot and GEPIA3 on 22/01/2026. Note: One gene, PRRC2C, is not available for analysis in KM-Plot.

Stress granules are not solely composed of G3BP2. Yang et al. [13] identified a seven-gene “key” component and a 36-gene “core” element of SGs [13]. We therefore assessed the potential survival benefits associated with either the “key” or “core” gene signatures for potential survival benefit. OS and PFS are presented in Figure 7.

Correlations between GB2 expression and each member of this core signature were assessed individually, and the results are presented in Table 2.

### 3.3. In Silico Demographic Clinicopathological Analyses in NSCLC Based off TCGA and CPTAC Datasets

To determine if there were any associations between G3BP2 expression and pathological characteristics, we examined the mRNA (TCGA) and proteomic (CPTAC) datasets using UALCAN [43]. Within the LUAD subtype, significant associations were found in several elements such as age, stage, gender and p53 mutational status as shown in Table 3.

In contrast, only a limited number of features showed any significant differences in the LUSC datasets, mostly in the areas of p53 mutational status as shown in Table 4.

### 3.4. Correlations Between G3BP2 mRNA Expression, Copy Number Alterations and Mutations in NSCLC

**Figure 8:**
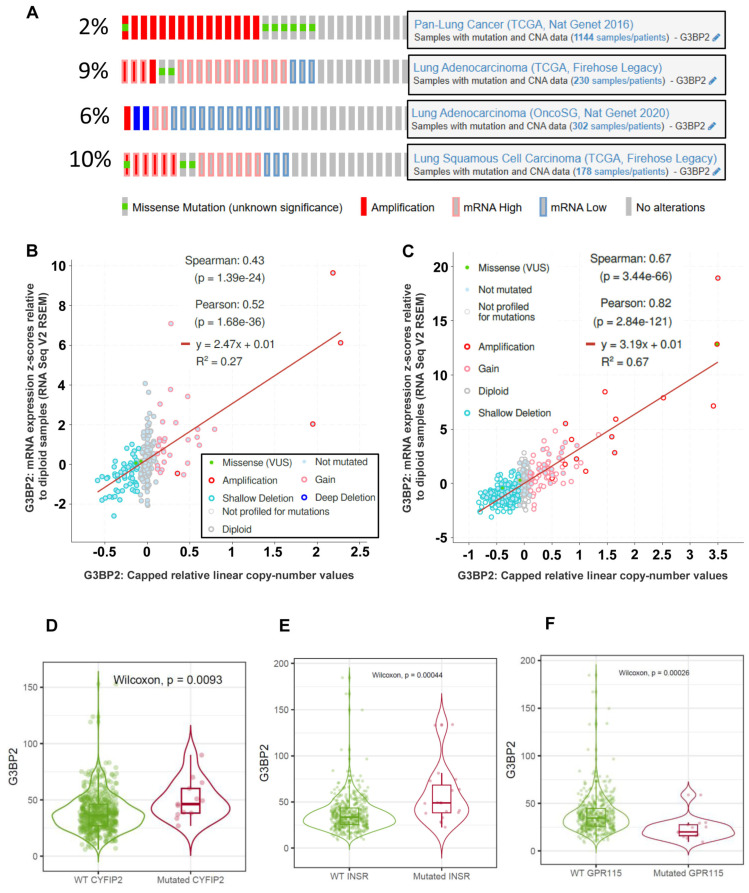
Analysis of CNA and mutations on G3BP2 expression in NSCLC. Levels of alterations for G3BP2 were examined in various datasets using cBioPortal as shown in (**A**). cBioPortal analysis identified positive correlations between CNA and mutations in (**B**) LUAD and (**C**) LUSC. Mutated genes which are correlated with altered G3BP2 mRNA expression were identified using muTarget and validated using TIMER3. Significant mutated genes identified were (**D**) CYFIP2, (**E**) INSR and (**F**) ADGRF4 (GPR115).

As G3BP2 is significantly dysregulated in NSCLC, to further study the potential effects of this dysregulation, we then utilized cBioPortal [36] to determine if there were any correlations between mutations or copy number variations (CNVs) and gene expression changes in the TCGA-LUAD and -LUSC datasets. As shown in Figure 8A, the proportion of samples with alterations range between 2 and 10% depending on the dataset analyzed. When reanalyzed for mRNA vs. copy number alterations (CNA), positive correlations between CNA and G3BP2 gene expression were observed in both the TCGA-LUAD (Figure 8B Spearman’s Rho: 0.43, *p* = 1.39 × 10^−24^) and the TCGA-LUSC datasets (Figure 8C, Spearman’s Rho: 0.67, *p* = 3.44 × 10^−66^). Both these data sets are predominantly Caucasian, but unfortunately it was not possible to conduct a similar analysis using the same parameters in the TCGA-OncoSG (Asian) dataset (in this instance we utilized capped relative linear copy-number values, which is not an available option for the OncoSG dataset).

We then examined for genes which, if mutated, were associated with changes in G3BP2 mRNA expression. A first analysis was conducted using MuTarget for a mutation prevalence of 2% (the default setting) [40]. This identified two genes in the LUAD dataset (CYFIP2 and PRKG2) and three genes in LUSC (INSR, FOXD4L5 and ADGRF4/GPR115) which, if mutated, had significantly altered expression of G3BP2. However, when these genes were re-analyzed in TIMER3 [39], only CYFIP2, INSR and ADGRF remained significant as shown in Figure 8D–F. This may, however, relate to the statistical test used by each platform, as MuTarget utilizes a Mann–Whitney U Test [40] while TIMER3.0 uses a Wilcoxon Test [39]. The results of both analyses are presented in Table 5.

### 3.5. Correlations Between G3BP2 mRNA Expression and Tumor Mutational Burden (TMB) in NSCLC

Using TIMER3 [39], key genes often associated in NSCLC as having oncogenic driver mutations were assessed to determine whether mutations within these key genes correlated, per se, with altered G3BP2 expression levels. The results are presented in Table 6.

Of these, only mutated p53 was found to alter expression of G3BP2, but only in LUSC. In contrast, positive correlations between G3BP2 mRNA expression and wild-type mRNA expression for most of these genes were observed for both LUAD and LUSC (Table 6), while a positive correlation between G3BP2 and ALK mRNA expression was observed only in the TCGA-LUAD samples (Table 6). These results suggest that oncogenic driver mutations do not play a role in the overexpression of G3BP2 mRNA in NSCLC.

**Figure 9:**
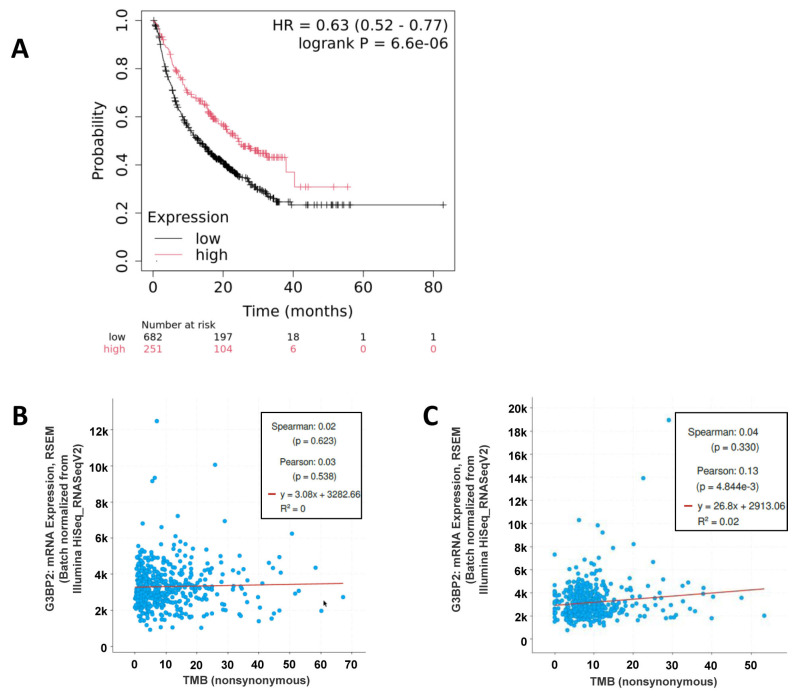
Correlations between G3BP2 mRNA expression response to immunotherapy and TMB. (**A**) KM-Plot analysis of a pan-cancer cohort demonstrates that high G3BP2 mRNA expression is associated with better OS. (**B**) There is no apparent association between GBP2 mRNA and TMB in LUAD; and (**C**) LUSC.

Tumor mutational burden (TMB) is traditionally associated as a potential biomarker for predicting the efficacy of immune checkpoint inhibitor (ICI) therapy [44], although this has recently been called into question [45]. We therefore examined if expression of G3BP2 mRNA was associated with OS benefit from ICI in a pan-cancer dataset. This analysis demonstrated that high expression of G3BP2 mRNA is associated with significantly better survival in patients undergoing ICI therapy (Figure 9A).

We then assessed if there were any correlations between G3BP2 mRNA expression and the key current targets for ICI therapy (namely PD-1, PD-L1 and CTLA-4) using TIMER3. Our analysis identified that only PD-L1 was significantly associated with G3BP2 expression (Table 7).

A proxy for assessing potential TMB is to analyze expression of a subset of genes associated with either DNA Damage Response (DDR) or Mismatch Repair (MMR) [21,46]. The results are presented for this in Table 8.

This association is, however, unlikely to be due to TMB as only weak correlations between TMB and G3BP2 mRNA expression were identified using cBioPortal for both LUAD (Figure 9B) and LUSC (Figure 9C).

### 3.6. CpG Methylation at the G3BP2 Gene Is Associated with Survival

An examination of G3BP2 expression using cBioPortal found an inverse association between G3BP2 mRNA expression and DNA methylation in LUAD (Spearman: −0.15; *p* = 8.987 × 10^−4^; n = 456; Supplementary Figure S2) and a similar inverse association in LUSC (Spearman: −0.37; *p* = 2.24 × 10^−13^; n = 370, Supplementary Figure S3), suggesting that increased DNA methylation may be associated with decreased G3BP2 mRNA expression. However, analysis of CpG promoter methylation using UALCAN suggests that overall, the promoter is hypomethylated in LUAD (Beta Value Max 0.183 − Min 0.09; Supplementary Figure S4) and LUSC (Beta value Max 0.168 − Min 0.07; Supplementary Figure S5). To put this in context, the beta value cut-off for hypomethylation in UALCAN is [Beta-value: 0.3 − 0.25] [43].

**Figure 10:**
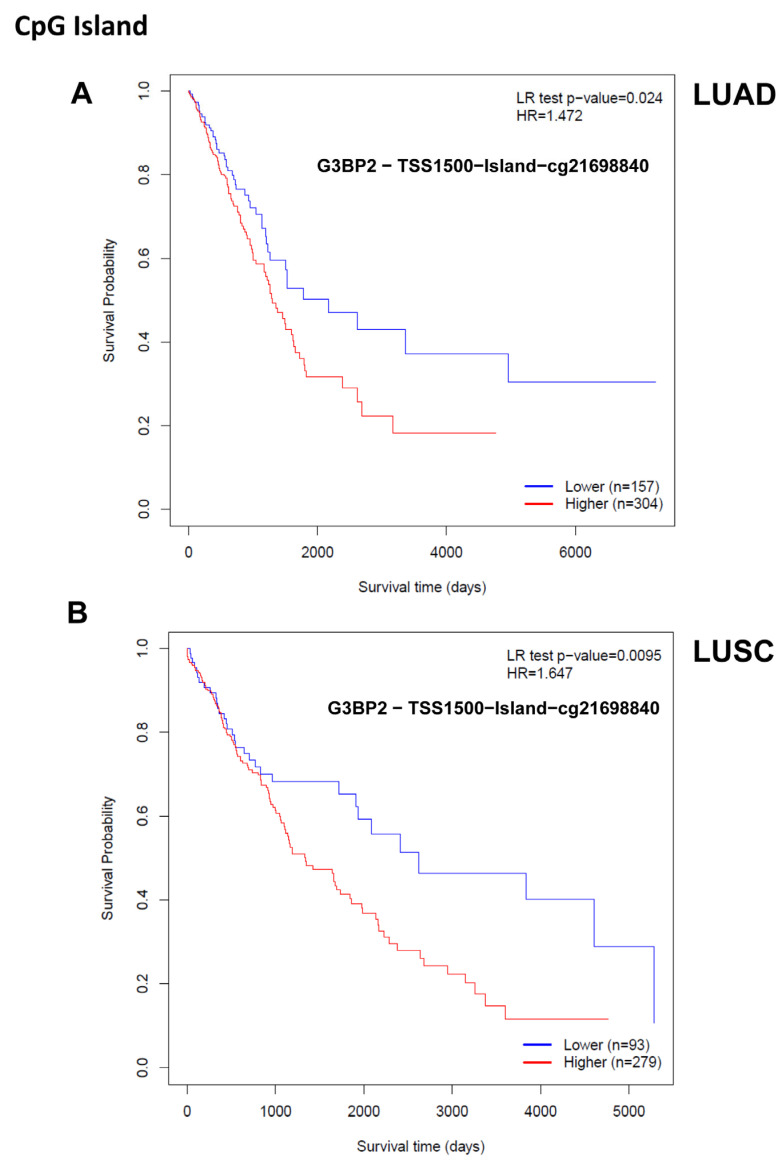
Methylation of a single CpG residue in the G3BP2 TSS is associated with OS. Increased methylation at residue cg21698840 is associated with worse OS in both (**A**) LUAD and (**B**) LUSC as determined using MethSurv. Analysis conducted/confirmed on 28 January 2026.

Nevertheless, to assess if DNA methylation at the G3BP2 locus was associated with patient OS we subsequently conducted an analysis of the TCGA-LUAD and -LUSC datasets using MethSurv [37]. From this analysis, the majority of CpG residues examined had no association with patient OS; however, increased methylation of one CpG residue (cg21698840) (at a position within 1500 of the transcriptional start site TSS) was found to have worse OS for both LUAD (*p* = 0.024) and LUSC (*p* = 0.0095) as shown in Figure 10.

### 3.7. Effects of Compound C108 on Cellular Proliferation

**Figure 11:**
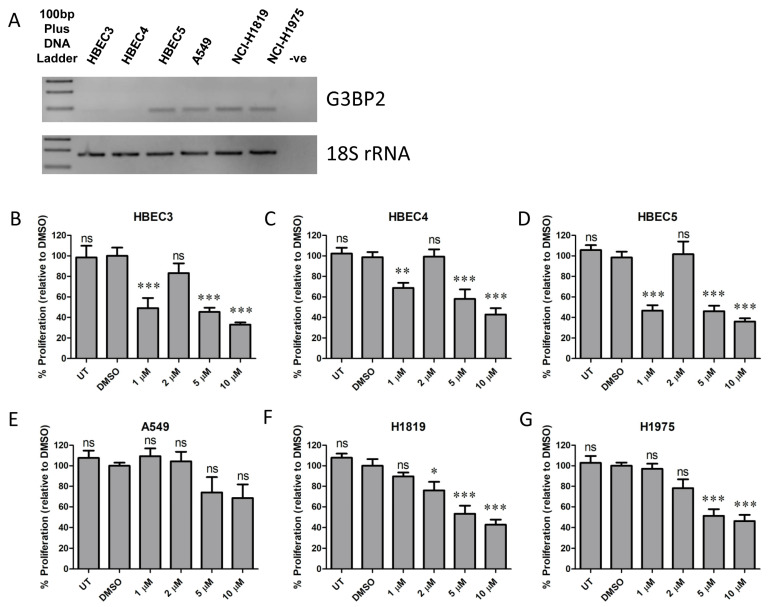
Effect of Compound C108 on cellular proliferation. Effects of the G3BP2 targeting agent Compound C108 were examined by (**A**) assessing the expression of G3BP2 mRNA, which was examined by endpoint PCR in a panel of normal human bronchial epithelial and NSCLC cell lines, followed by assays examining its effects on cellular proliferation in normal human bronchial epithelial cells (HBECs) HBEC3 (**B**), HBEC4 (**C**) and HBEC5 (**D**). These were compared to effects on proliferation in the NSCLC cell lines A549 (**E**), NCI-H1819 (**F**) and NCI-H1975 (**G**). Concentrations used were based off the data [47]. The data are expressed as the mean  ±  SEM of three biological replicates. ns—not significant; *—*p* < 0.05; **—*p* < 0.01; ***—*p* < 0.001.

Compound C108 has been identified as a chemical with the ability to bind and inhibit G3BP2 [22]. As G3BP2 could potentially be a candidate target for therapy in NSCLC, we examined the expression of G3BP2 mRNA in a panel of cell lines as shown in Figure 11.

The results of this show that while Compound C108 has anti-proliferative effects on NSCLC cell lines at high concentrations (Figure 11F,G), some cancer cell lines such as A549 are insensitive to this drug (e.g., Figure 11E). Moreover, normal human bronchial epithelial cells (HBECs), even with limited mRNA expression of G3BP2 (Figure 11A, Supplementary Figure S7), appear to be extremely sensitive to Compound C108. All HBECs showed a biphasic response to this agent and, at low concentration (1 µM), were significantly affected (Figure 11B–D). Critically, this concentration had no effect on any NSCLC cell line tested (Figure 11E–G).

## 4. Discussion

Under conditions of cellular stress, condensation of stress granules (SGs) occurs through the action of proteins G3BP1 and G3BP2 (G3BPs), resulting in a massive reduction in protein translation [13,48]. The role of SGs in cancer is an emerging area of clinical significance [12].

In lung cancer, G3BP1 has been identified as an overexpressed gene associated with poor prognosis in NSCLC [15]. Likewise, an early study by Li et al. demonstrated that G3BP2 was overexpressed at the protein level in NSCLC [19], while links between G3BP2 and another core SG component (HDAC6) in lung cancer have been identified [18]. To our knowledge, there has been no comprehensive analysis of the prognostic value of G3BP2 in NSCLC. We originally demonstrated that USO1, a gene located in the same cytogenetic location, had prognostic value in NSCLC [21]. USO1 is straddled on one side by G3BP2 and on the other side by PPEF2. An initial assessment for potential OS benefit using KM-Plot identified G3BP2 as having potential OS prognostic value (*p* = 8.4 × 10^−6^) whilst PPEF2 did not (*p* = 0.15).

Given that G3BP2 was identified as being overexpressed in NSCLC [19], we first examined G3BP2 expression in a small cohort of surgically resected fresh-frozen samples (Figure 1A), which suggested that G3BP2 mRNA is not overexpressed. However, a subsequent analysis of the CPTAC datasets (Figure 1B,D) show that G3BP2 mRNA is indeed significantly upregulated in both NSCLC lung adenocarcinoma (LUAD) and lung squamous cell carcinoma (LUSC) subsets [31]. Moreover, the overexpression of G3BP2 was also reflected at the protein level in the same samples of the CPTAC datasets (Figure 1C,E) [31]. The difference in mRNA expression observed between our samples and the CPTAC may simply reflect differences in sample size.

The CPTAC proteomic data shows increased phosphorylation at Serines S141 and S149 (Supplementary Figure S1). In this regard, an early paper reported that phosphorylation at S149 was key to the role of G3BP2 mediated SG formation [49]. However, it must be noted that some of the data supporting this has since been identified as being unreliable [49], and whilst this may suggest enhanced SG formation in NSCLC by G3BP2, a degree of caution is advised.

Given the general overexpression of G3BP2 in NSCLC and its potential prognostic value, we conducted an IHC analysis of G3BP2 on an in-house tissue microarray. Our results showed that overall (i.e., in all histologies), there was a trend towards better OS for higher expression of G3BP2 (Figure 3A), but it remained non-significant when stratified for either LUAD (Figure 3B) or LUSC (Figure 3C). In silico analyses using KM-Plot confirmed that high G3BP2 mRNA expression was associated with both OS (Figure 4A) and PFS (Figure 4D) benefit for all histologies. Moreover, this appears to be associated primarily with LUAD OS (Figure 4B) and PFS (Figure 4D), with no significant OS or PFS benefit for LUSC (Figure 4C,F). However, a reanalysis of the TCGA datasets using GEPIA3 did not see any OS (Figure 5A) or PFS benefit (Figure 5B). This may reflect the differences between the data used. KM-Plot analyses utilized microarray data for one specific probe (208841_s_at), while GEPIA3 analyses NGS sequencing data. As such, the probe used in KM-Plot may miss different mRNA isoforms. This may be reflected in our analysis of the TCGA datasets using OncoSplicing as two alternative promoters were identified whose expression (mapped as percentage splice in or PSI) showed very different OS benefit for both LUAD and LUSC datasets (Figure 6).

G3BP2 does not, however, act by itself when SGs form. A core network of additional genes/proteins have been identified [13] which are central to SG function. We therefore examined associations between these genes and G3BP2 and showed that all genes were positively associated at high significance in both LUAD and LUSC with G3BP2 mRNA (Table 2). In a similar manner to the observations observed for G3BP2, high expression of this core signature was associated with better OS (but not PFS) when assessed using KM-Plot (Figure 7A,C), but no survival benefit was associated with this signature when the TCGA datasets were tested using GEPIA3 (Figure 7B,D). Again, this may reflect either differences in the makeup of each analysis (microarray versus NGS), or it may reflect numbers included in each. KM-Plot microarray patient numbers were (n = 1411) for OS and (n = 874) for PFS analyses, whereas GEPIA3 NGS analyses had (n = 996) for both OS and PFS. The discrepancies between the individual analyses suggest that at the present time, more work will be required to definitively link G3BP2 expression (mRNA or protein) as a potential prognostic biomarker in NSCLC.

In terms of patient demographic, clinicopathological analyses as presented in Table 3 and Table 4 identify significant links in various categories (e.g., age, etc.) which are predominantly significant in the LUAD datasets. Of particular interest, G3BP2 expression is significantly different when the mutational status of TP53 is considered (Table 3 and Table 4). Given the proportion of alterations common to NSCLC (Figure 8A), the positive correlations between copy number aberrations (CNA) and G3BP2 mRNA expression in both LUAD (Figure 8B) and LUSC (Figure 8C), and the known significance of oncogenic driver mutations in NSCLC [50], we also assessed whether key oncogenic driver mutations were associated with altered G3BP2 mRNA expression (Table 6). While only TP53 remained significant, suggesting that oncogenic driver mutations may not be directly linked to SG formation in NSCLC, links between KRAS and G3BP1 and SGs have been associated with enhanced tumor survival in colon and pancreatic cancer settings [51]. We also conducted an analysis using muTarget to identify other novel genes (Table 5) which, if mutated, were associated with altered G3BP2 mRNA expression as shown in Figure 8D–F. Three candidates were identified. Cyfip2 (Figure 8D) has no known interactions with G3BP2 but has been associated with SG formation in HeLa cells [52]. The next candidate is the insulin receptor (InsR), and it also has been linked to SG formation under cellular stress [53]. The last novel gene identified is ADGRF4 (also known as GPR115) (Figure 8F), and while no links between this gene and SG have yet been identified, potential roles for this receptor in NSCLC have been identified [54,55,56].

Given the importance of immunotherapy in NSCLC, the potential for G3BP2 expression to predict response was assessed using gene expression and a proxy subset of genes associated with either the DNA Damage Response (DDR) or Mismatch Excision Repair (MMR) [21,46]. Correlations between G3BP2 mRNA expression and the key immune checkpoint targets currently approved for use in NSCLC identified only a correlation between PD-L1 (CD274) and G3BP2 mRNA (Table 7). Significant correlations between G3BP2 mRNA and DNA Damage Response (DDR) or Mismatch Excision Repair (MMR) genes were observed (Table 8), but an assessment of TMB and G3BP2 mRNA expression in the TCGA datasets using cBioPortal did not find any positive correlations between TMB and G3BP2 in either LUAD (Figure 9B), or LUSC (Figure 9C), despite a pan-cancer analysis using KM-Plotter [38], suggesting that high G3BP2 mRNA predicts a better probability of response to immunotherapy (Figure 9A) overall in cancer.

Given that aberrant DNA methylation is a common feature in NSCLC [57], we also examined if there were any links between G3BP2 and DNA CpG methylation. While initial analysis suggests that methylation of the G3BP2 promoter may be associated with decreased mRNA expression (Supplementary Figures S2 and S3), it would appear that overall, the promoter of G3BP2 is predominantly hypomethylated (Supplementary Figures S4 and S5). Nevertheless, we examined whether any CpG residues within the G3BP2 promoter had any potential prognostic value using MethSurv [37]. From this analysis, a single CpG residue was identified where higher methylation was associated with significantly worse OS in both LUAD (Figure 10A) and LUSC (Figure 10B). Whether this reflects decreased G3BP2 mRNA expression in these samples is unknown, while its potential for diagnostic or routine testing remains unclear. Moving forward, more work will be required to unravel the potential of this epigenetic mark in this regard.

The potential to therapeutically target G3BP2 and/or SGs is an emerging area of increasing medical interest [22,47,58]. In this regard, the chemical Compound C108 has been shown to interfere with SG granule formation by its interaction with G3BP2 [22]. We examined whether this compound had any potential for use in NSCLC. Proliferation assay results as presented in Figure 11 suggest that targeting G3BP2 would not be advisable as normal bronchial epithelial cells are more sensitive to this compound than cancer cells. However, one caveat is that the conditions used for this analysis may not truly reflect stressed cells, and in the future, additional experiments may be required to assess if conditions that more accurately reflect stressed conditions provide more granularity. In this regard, targeting SGs may have a better role in re-sensitizing cancer cells to therapy. For example, G3BP2 targeting may only be effective in cells being stressed by chemotherapy in a manner such as that observed by Gupta et al. [22], or through combining different targeting agents such as HDAC6 inhibitors [18]. There is additional evidence that G3BP2 and SGs play a role in the response to cisplatin in triple-negative breast cancer (TNBC), in that depletion of G3BP2 promotes cell-cycle arrest in cisplatin-resistant cell lines and sensitizes cisplatin-resistant TNBC cell lines to cisplatin [59]. We assessed the expression of G3BP2 in a panel of isogenic parent/resistant NSCLC cells. Our results show that the expression of G3BP2 is not altered between parent/resistant cells (Supplementary Figure S6), indicating that an alternative mechanism may play a role. This may indeed be the case as Heijink et al. demonstrated that G3BP2 promotes cell death in sensitive cells but paradoxically inhibits cell death in resistant cells [59]. As such, further evaluation of the potential for combining G3BP2 targeting agents with cisplatin will be required moving forwards. It may also be possible to combine targeting SGs to improve immunotherapy. There is one publication showing that Compound C108 increased CD8 T-cell proliferation and infiltration in breast and glioblastoma tissue due to decreased PD-L1 expression [41]. Indeed, given the links between G3BP2 and HDAC6, and the clinical interest in the use of HDAC6 inhibitors to potentiate/improve immunotherapy [60,61,62], this remains a potentially important therapeutic avenue for future exploration. However, the caveat still remains regarding the higher sensitivity of normal bronchial epithelial cells to these agents, but these possibilities remain to be explored.

There are a number of outstanding issues that limit the scope of this current study that warrant further investigation. For example, the disparities between analyses from various datasets suggests that key data may be lacking (for example, if microarray probes miss splice variants or different mRNA isoforms compared to NGS sequencing). Moreover, the analyses and options available currently cannot yet correlate survival with oncogene-driven vs. non-oncogene-driven NSCLC. Given that G3BP2 has been reported to inhibit PD-L1 expression [41], this molecular stratification will be critical for understanding its role in different NSCLC subtypes. Indeed, the expression shown for OS benefit in Figure 9A had to be limited to a pan-cancer analysis as the numbers of available NSCLC could not be run alone due to “insufficient sample size for statistical analysis”.

Likewise, the issue also arises of whether or not G3BP2 should be assessed solely as SGs by IHC. In this instance, we believe that to truly ascertain if the granularity observed in our IHC is truly an SG, it would require a dual stain for another SG member (for example, G3BP1 or HDAC6), and our current analysis is based solely off total intensity and distribution only.

Functional evaluation of G3BP2 targeting compounds as currently presented are limited as it focuses on baseline expression and proliferation after treatment with G3BP2 targeting agent. The actual roles of G3BP2 in stress granules, DNA Damage Response, and chemoresistance remain to be directly tested, for example, using apoptosis assay, stress granule formation, and drug sensitivity assays. The results would strengthen the conclusions regarding potential mechanisms involved. In this regard, it remains unclear whether the observed effects are specifically due to G3BP2, and future experiments will require validation through knockdown (e.g., siRNA/CRISPR/Degron) or rescue approaches. These are important, but it must also be noted that the effects of Compound C108 particularly with respect to normal bronchial epithelial cells (Figure 11A–D) suggest that off-target events may be occurring and that careful consideration regarding this agent is warranted. Given the sensitivity of normal bronchial cells to C108, if targeting SGs is a valid approach for re-sensitizing cells to chemotherapy, it may be necessary to target it in another fashion, for example, a HDAC6i such as ricolinostat, which has entered clinical Phase I/II settings, may be a better approach.

## 5. Conclusions

Stress granules are emerging as significant players in cancer. Achieving a better understanding of their roles and activities in cancer will be required to assess or develop strategies to better target this pathway in a clinical setting. Two key genes/proteins, G3BP1 and G3BP2, have emerged as potential central players in SG formation and biological function. While SG biology has been well characterized in several cancers including breast and pancreatic cancer, the data for NSCLC is less well-developed.

In this manuscript, we endeavored to conduct a comprehensive analysis of one of these key players, G3BP2, in NSCLC. Our analyses link the expression of this gene across the continuum of NSCLC to encompass general overexpression, survival benefit, mutational burden and oncogenic drivers, and assessment of cellular health in response to directly targeting this protein and examine links to its potential to improve responses to immunotherapy. The results suggest that while strong links between G3BP2 and many clinical parameters in NSCLC exist, targeting SGs through G3BP2 may not be suitable as a single agent approach. Moving forward, more work will be required to full delineate if combined therapies may have better potential clinical efficacy in NSCLC or other cancer models.

## Figures and Tables

**Table 1:** Details of surgically resected fresh-frozen patient samples used in this study.

Sample	Histology	Sex	Age (At Surgery)	Stage (7th Edition)	TNM
1	Adenocarcinoma	Female	61	IB	pT2a N0 IB
2	Adenocarcinoma	Female	64	IB	pT2a N0
3	Adenocarcinoma	Female	67	IB	pT2a N0
4	Adenocarcinoma	Female	74	IB	pT2a N0
5	Adenocarcinoma	Female	67	IIA	pT2a N1
6	Adenocarcinoma	Female	61	IIB	pT2b N2
7	Adenocarcinoma	Female	55	IIIA	N/A
8	Adenocarcinoma	Male	64	IIIA	pT2a N0 Mx
9	Adenocarcinoma	Female	71	IV	pT4 N2 M1a
10	Adenocarcinoma	Female	55	IIIA	pT4 N1
11	Squamous Cell Carcinoma	Female	76	IA	T1b N0
12	Squamous Cell Carcinoma	Female	71	IB	pT2a N0
13	Squamous Cell Carcinoma	Male	77	IB	pT2a N0
14	Squamous Cell Carcinoma	Male	68	IB	pT2a
15	Squamous Cell Carcinoma	Male	69	IIA	pT2a N1
16	Squamous Cell Carcinoma	Male	62	IIA	pT1b N1
17	Squamous Cell Carcinoma	Male	57	IIA	pT2a N1
18	Squamous Cell Carcinoma	Female	68	IIA	pT2b N0
19	Squamous Cell Carcinoma	Female	70	IIA	pT2a N0
20	Squamous Cell Carcinoma	Female	57	IIA	pT2a N1 Mx

**Table 2:** Correlations between key SG core network genes and G3BP2 in LUAD and LUSC.

		LUAD		LUSC	
	Variable	R	*p*-Value	R	*p*-Value
Key SG Assembly genes	CSDE1	0.595	1.52 × 10^−50^ ***	0.375	4.12 × 10^−18^ ***
	G3BP1	0.62	6.62 × 10^−56^ ***	0.425	2.95 × 10^−23^ ***
	HDAC6	0.093	0.035 *	0.113	0.0115 *
	PRRC2C	0.453	1.94 × 10^−27^ ***	0.402	8.88 × 10^−21^ ***
	TIA1	0.082	0.0691	0.233	1.45 × 10^−7^ ***
	UBAP2L	0.197	6.49 × 10^−6^ ***	0.205	3.82 × 10^−6^ ***
	Combined Assembly Signature	0.449	5.88 × 10^−27^ ***	0.368	2.23 × 10^−17^ ***
Additional “Constituent” SG genes	ATXN2	0.178	5 × 10^−5^ ***	0.252	1.24 × 10^−8^ ***
	ATXN2L	0.0366	0.408	0.107	0.0167 *
	CAPRIN1	0.538	5.23 × 10^−40^ ***	0.389	2.05 × 10^−19^ ***
	CBX1	0.391	3.28 × 10^−20^ ***	0.35	9.33 × 10^−16^ ***
	DDX19A	0.481	4.02 × 10^−31^ ***	0.381	1.2 × 10^−18^ ***
	DDX3X	0.6	9.27 × 10^−52^ ***	0.504	2.23 × 10^−33^ ***
	EIF3D	0.158	0.000314 ***	0.156	0.000489 ***
	EIF3E	0.145	0.000967 ***	0.132	0.00326 ***
	EIF3G	0.0616	0.163	0.0104	0.816
	EIF3I	0.18	3.9 × 10^−5^ ***	0.219	7.75 × 10^−7^ ***
	FMR1	0.364	1.29 × 10^−17^ ***	0.339	6.85 × 10^−15^ ***
	HNRNPA2B1	0.416	6.11 × 10^−23^ ***	0.277	3.33 × 10^−10^ ***
	KPNB1	0.471	7.86 × 10^−30^ ***	0.425	2.73 × 10^−23^ ***
	MACF1	0.359	3.78 × 10^−17^ ***	0.277	3.39 × 10^−10^ ***
	NUFIP2	0.593	3.18 × 10^−50^ ***	0.464	5.38 × 10^−28^ ***
	NUP98	0.458	4.69 × 10^−28^ ***	0.38	1.61 × 10^−18^ ***
	NXF1	−0.138	0.00166 **	0.027	0.548
	POLR2B	0.67	9.6 × 10^−65^ ***	0.686	1.23 × 10^−70^ ***
	PPP1R10	0.26	2.14 × 10^−9^ ***	0.311	1.33 × 10^−12^ ***
	PPP2R1A	0.131	0.00299 **	0.205	4.19 × 10^−6^ ***
	RAB1A	0.469	1.7 × 10^−29^ ***	0.288	5.79 × 10^−11^ ***
	SRSF3	0.494	4.52 × 10^−33^ ***	0.429	9.27 × 10^−24^ ***
	TAF15	0.292	1.35 × 10^−11^ ***	0.269	1.12 × 10^−9^ ***
	TIAR (TIAL1)	0.305	1.59 × 10^−12^ ***	0.124	0.00553 **
	TRIM25	0.401	2.41 × 10^−21^ ***	0.446	1.15 × 10^−25^ ***
	TRIM56	0.374	1.46 × 10^−18^ ***	0.249	1.88 × 10^−8^ ***
	UBAP2	0.304	1.86 × 10^−12^ ***	0.18	5.48 × 10^−5^ ***
	UPF1	0.218	5.81 × 10^−7^ ***	0.215	1.25 × 10^−6^ ***
	USP10	0.466	4.53 × 10^−29^ ***	0.364	5.06 × 10^−17^ ***
	Combined Signature	0.535	1.57 × 10^−39^ ***	0.454	1.17 × 10^−26^ ***
Combined “Core” SG Network	Combined “core” Signature	0.534	2.48 × 10^−39^ ***	0.449	4.31 × 10^−26^ ***

Analysis was conducted using GEPIA3. Results are presented as Spearman’s rho value (R) alongside statistical significance. *—*p* < 0.05; **—*p* < 0.01; ***—*p* < 0.001. Data generated on 2 December 2024 via GEPIA2 and reanalyzed on GEPIA3 on 14 January 2026.

**Table 3:** Demographic clinicopathological analyses for LUAD based off TCGA and CPTAC datasets.

Demographic	mRNA	Protein
Stage	Normal vs. Stage 1: *p* = 0.08 Normal vs. Stage 2: *p* = 0.0044 ** Normal vs. Stage 3: *p *= 0.036 * Normal vs. Stage 4: *p* = 0.057 (All other combinations not significant)	Normal vs. Stage 1: *p *= 5.4 × 10^−22^ *** Normal vs. Stage 2: *p *= 1.08 × 10^−13^ *** Normal vs. Stage 3: *p *= 2.9 × 10^−12^ *** Normal vs. Stage 4: N/A (All other combinations not significant)
Race	Normal vs. Caucasian: *p *= 0.0008 *** Normal vs. African American: *p* = 0.762 Normal vs. Asian: *p* = 0.727 (All other combinations not significant)	Normal vs. Caucasian: *p* = 1.67 × 10^−12^ *** Normal vs. African American: N/A Normal vs. Asian: N/A
Gender	Normal vs. Male: *p *= 0.0022 * Normal vs. Female: *p *= 0.0166 * Male vs. Female: *p* = 0.288	Normal vs. Male: *p *= 5.32 × 10^−28^ *** Normal vs. Female: *p *= 1.27 × 10^−18^ *** Male vs. Female: *p* = 0.389
Age	Normal vs. Age (21–40 Years): *p* = 0.67 Normal vs. Age (41–60 Years): *p* = 0.319 Normal vs. Age (61–80 Years): *p *= 0.00142 ** Normal vs. Age (81–100 Years): *p *= 0.0115 * Age (41–60 Yrs) vs. Age (81–100 Yrs): *p *= 0.0064 ** (All other combinations not significant)	Normal vs. Age (21–40 Years): *p* = 0.1314 Normal vs. Age (41–60 Years): *p *= 2.484 × 10^−20^ *** Normal vs. Age (61–80 Years): *p* = 9.76 × 10^−25^ *** Normal vs. Age (81–100 Years): *p* = 0.202 (All other combinations not significant)
Smoking	Normal vs. non-smoker: *p* = 0.073 Normal vs. smoker: *p* = 0.101 Normal vs. reformed smoker (<15 years): *p *= 0.0134 Normal vs. reformed smoker (>15 years): *p *= 0.00544 (All other combinations not significant)	No data available
TP53 mutational status	Normal vs. TP53-Mutant: *p *= 0.0006185 *** Normal vs. TP53-Non-Mutant: *p *= 0.0472 * TP53-Mutant vs. TP53-Non-Mutant *p* = 0.0633	Normal vs. p53/Rb-related pathway altered: *p *= 1.02 × 10^−35^ *** Normal vs. Others: *p *= 4.08 × 10^−7^ *** p53/Rb-related pathway altered vs. Others: *p* = 8.67 × 10^−3^ ***

Analysis was conducted using UALCAN. Results are presented as Spearman’s rho value (R) alongside statistical significance. *—*p* < 0.05; **—*p* < 0.01; ***—*p* < 0.001. Data generated on 27 November 2024.

**Table 4:** Demographic clinicopathological analyses for LUSC based off TCGA and CPTAC datasets.

Demographic	mRNA	Protein
Stage	Normal vs. Stage 1: *p* = 0.4011 Normal vs. Stage 2: *p* = 0.1477 Normal vs. Stage 3: *p* = 0.1931 Normal vs. Stage 4: *p* = 0.1539	No data available
Race	Normal vs. Caucasian: *p* = 0.1327 Normal vs. African American: *p* = 0.032 * Normal vs. Asian: *p* = 0.727 (All other combinations not significant)	No data available
Gender	Normal vs. Male: *p* = 0.4348 Normal vs. Female: *p* = 0.5620 Male vs. Female: *p* = 0.9911	Normal vs. Male: *p *= 4.34 × 10^−29 ^*** Normal vs. Female: *p *= 2.61 × 10^−11^ *** Male vs. Female: *p* = 0.0920
Age	Normal vs. Age (21–40 Years): *p* = 0.659 Normal vs. Age (41–60 Years): *p* = 0.57 Normal vs. Age (61–80 Years): *p* = 0.417 Normal vs. Age (81–100 Years): *p* = 0.366 (All other combinations not significant)	No data available
Smoking	Normal vs. non-smoker: *p* = 0.205 Normal vs. smoker: *p* = 0.2375 Normal vs. reformed smoker (<15 years): *p* = 0.924 Normal vs. reformed smoker (>15 years): *p* = 0.7612 (All other combinations not significant)	No data available
TP53 mutational status	Normal vs. TP53-Mutant: *p* = 0.1009 Normal vs. TP53-Non-Mutant: *p *= 0.0195 ** TP53-Mutant vs. TP53-Non-Mutant *p *= 0.00014 ***	Normal vs. p53/Rb-related pathway altered: *p *= 2.58 × 10^−35 ^*** Normal vs. Others: 0.1452 p53/Rb-related pathway altered vs. Others: *p* = 0.2294

Analysis was conducted using UALCAN. Results are presented as Spearman’s rho value (R) alongside statistical significance. *—*p* < 0.05; **—*p* < 0.01; ***—*p* < 0.001. Data generated on 27 November 2024.

**Table 5:** Mutated genes identified using MuTarget and TIMER3.

Gene	MuTarget	TIMER3	Cancer
CYFIP2	*p* = 0.00911 **	*p* = 0.0093 **	LUAD
PRKG2	*p* = 0.00315 **	Not significant	LUAD
INSR	*p* = 0.000281 ***	*p* = 0.00044 ***	LUSC
FOXD4L5	*p* = 0.00203 **	Not Found	LUSC
ADGRF4 (GPR115)	*p* = 0.00261 **	*p* = 0.00026 ***	LUSC

Analysis was conducted using MuTarget and TIMER3. **—*p* < 0.01; ***—*p* < 0.001. Data generated on 27 January 2026.

**Table 6:** Correlations between mutated versus non-mutated oncogenic drivers in G3BP2 mRNA expression.

	Mutated	Non-Mutated	Mutated	Non-Mutated
Gene	LUAD		LUSC	
TP53	*p* = 0.948	*p* = 0.015 *	*p* = 0.00491 **	*p* = 0.0018 **
KRAS	*p* = 0.29	*p* = 5 × 10^−30^ ***	*p* = 0.24	*p* = 4.9 × 10^−11^ ***
EGFR	*p* = 0.08	*p* = 7.11 × 10^−23^ ***	*p* = 0.19	*p* = 4.3 × 10^−8^ ***
ERBB2	*p* = 0.33	*p* = 3.11 × 10^−6^ ***	*p* = 0.57	*p* = 6.49 × 10^−6^ ***
PIK3CA	*p* = 0.44	*p* = 2.72 × 10^−51^ ***	*p* = 0.43	*p* = 3.09 × 10^−21^ ***
ALK	*p* = 0.085	*p* = 2.28 × 10^−5^ ***	*p* = 0.40	*p* = 0.233
ROS1	*p* = 0.41	*p* = 7.76 × 10^−7^ ***	*p* = 0.73	*p* = 0.45
NTRK1	*p* = 0.052	*p* = 0.03 *	*p* = 0.91	*p* = 0.64
NTRK2	*p* = 0.43	*p* = 0.00047 ***	*p* = 0.72	0.0003 ***
NTRK3	*p* = 0.2	*p* = 0.078	*p* = 0.45	*p* = 0.322
BRAF	*p* = 0.76	*p* = 0.61 × 10^−21^ ***	*p* = 0.74	*p* = 5.41 × 10^−14^ ***
FGFR1	*p* = 0.269	*p* = 0.03 *	*p* = 0.396	*p* = 3.85 × 10^−6^ ***
FGFR2	*p* = 0.18	*p* = 0.038 *	*p* = 0.54	*p* = 1.1 × 10^−7^ ***
TROP2	*p* = 0.51	*p* = 7.97 × 10^−5^ ***	*p* = 0.16	*p* = 0.047 *

Analysis was conducted using TIMER3. LUAD n = 512; LUSC: n = 482 patients. *—*p* < 0.05; **—*p* < 0.01; ***—*p* < 0.001. Data generated on 10 February 2026.

**Table 7:** Correlations between key ICI targets and G3BP2 mRNA expression.

Gene	LUAD	LUSC
PD-1 (PDCD1)	*p* = 0.28	0.43
PD-L1 (CD274)	*p* = 9.8 × 10^−11^ ***	*p* = 0.01 *
CTLA4	*p* = 0.94	*p* = 0.64

Analysis was conducted using TIMER3. *—*p* < 0.05; ***—*p* < 0.001. Data generated on 28 January 2026.

**Table 8:** Correlation between G3BP2 expression and proxy markers of TMB.

		LUAD		LUSC	
	Variable	R	*p*-Value	R	*p*-Value
DNA Damage Response (DDR) Pathway	ATM	0.426	4.32 × 10^−24^ ***	0.35	1.67 × 10^−15^ ***
	ATR	0.117	0.00081 ***	0.097	0.031 *
	CHEK1	0.238	4.88 × 10^−8^ ***	0.282	1.51 × 10^−10^ ***
	CHEK2	−0.0342	0.439	0.051	0.255
	BRCA1	0.317	1.65 × 10^−13^ ***	0.292	2.91 × 10^−11^ ***
	BRCA2	0.364	1.46 × 10^−17^ ***	0.278	2.87 × 10^−10^ ***
	TP53	0.16	0.000269 ***	0.141	0.00158 **
	PRKDC	0.346	5.84 × 10^−16^ ***	0.316	4.78 × 10^−13^ ***
	RAD51	0.203	3.56 × 10^−6^ ***	0.177	7.26 × 10^−5^ ***
	Combined Signature	0.3	3.35 × 10^−12^ ***	0.305	3.82 × 10^−12^ ***
Mis-match excision repair (MMR) related genes	MLH1	0.52	4.58 × 10^−37^ ***	0.394	5.5 × 10^−20^ ***
	MLH3	0.16	0.000368 ***	0.12	0.00727 **
	MSH2	0.471	8.1 × 10^−30^ ***	0.385	5.11 × 10^−19^ ***
	MSH3	0.635	1.47 × 10^−59^ ***	0.473	4.07 × 10^−29^ ***
	MSH6	0.559	1.22 × 10^−43^ ***	0.414	4.66 × 10^−22^ ***
	PMS1	0.33	1.6 × 10^−14^ ***	0.286	1.6 × 10^−10^ ***
	PMS2	0.484	1.42 × 10^−31^ ***	0.397	3.33 × 10^−20^ ***
	Combined Signature	0.619	7.65 × 10^−56^ ***	0.47	9.86 × 10^−29^ ***
DDR & MMR	Combined Signature	0.412	1.52 × 10^−22^ ***	0.394	6.81 × 10^−20^ ***

Results are presented as Spearman’s rho value (R) alongside statistical significance. *—*p* < 0.05; **—*p* < 0.01; ***—*p* < 0.001. Analysis originally conducted using GEPIA2 on 21 November 2024 and repeated on GEPIA3 on 28 January 2026.

## Data Availability

Publicly available datasets were analyzed in this study. These data can be accessed and may be interrogated via TIMER: https://cistrome.shinyapps.io/timer/ (last accessed on 19 January 2026), TIMER3.0: https://compbio.top/timer3/ (last accessed on 11 February 2026), GEPAI3.0: https://gepia3.bioinfoliu.com/ (last accessed on 11 February 2026), KM-PLOT: https://kmplot.com/analysis/index.php?p=home (last accessed on 19 January 2026), cBioPortal: https://www.cbioportal.org/ (last accessed on 19 January 2026), muTarget: https://www.mutarget.com/ (last accessed on 19 January 2026), cProSite: https://cprosite.ccr.cancer.gov/#/ (last accessed on 19 January 2026), Methsurv: https://biit.cs.ut.ee/methsurv/ (last accessed on 28 January 2026), OncoSplicing: http://www.oncosplicing.com/ (last accessed on 10 February 2026). Additional data presented in this study from patient samples are available on request from the corresponding author and are not publicly available due to privacy/GDPR restrictions.

## Supplementary Materials

The following supporting information can be downloaded at: https://www.mdpi.com/article/10.3390/cancers18060969/s1, Supplementary Figure S1. Phosphoproteomic analysis of G3BP2 from the CPTAC LUAD and LUSC cProSite datasets; Supplementary Figure S2. cBioPortal analysis of G3BP2 mRNA expression and DNA methylation in LUAD from the TCGA-LUAD dataset; Supplementary Figure S3. cBioPortal analysis of G3BP2 mRNA expression and DNA methylation in LUSC from the TCGA-LUAD dataset; Supplementary Figure S4. G3BP2 DNA CpG promoter methylation analysis of the from the TCGA-LUAD dataset using UALCAN; Supplementary Figure S5. G3BP2 DNA CpG promoter methylation analysis of the from the TCGA-LUSC dataset using UALCAN; Supplementary Figure S6. G3BP2 mRNA expression in a panel of isogenic parent-resistant NSCLC cell lines. Supplementary Figure S7. Original gel image used to generate Figure 11A.

## Abbreviations

The following abbreviations are used in this manuscript:

CNA	Copy number alterations
CPTAC	Clinical Proteomic Tumor Analysis Consortium
DDR	Damage response
DNA	Deoxyribonucleic acid
ER	Endoplasmic reticulum
FBS	Fetal Bovine Serum
HBEC	Human bronchial epithelial cells
H-Score	Histochemical Score
G3BP1	GTPase-activating protein-binding protein 1
G3BP2	GTPase-activating protein-binding protein 2
ICI	Immune checkpoint inhibitor
IHC	Immunohistochemistry
LUAD	Lung adenocarcinoma
LUSC	Lung squamous cell carcinoma
MMR	Mismatch Repair
mRNA	Messenger RNA
mRNP	Messenger ribonucleoproteins
NSCLC	Non-small cell lung cancer
OS	Overall survival
PFS	Progression-free survival
PSI	Percent splice in
RBP	RNA-binding protein
SCLC	Small cell lung cancer
SG	Stress granules
TCGA	The Cancer Genome Atlas
TIIC	Tumor-infiltrating immune cells
TNBC	Triple-negative breast cancer
TMA	Tissue microarray
TMB	Tumor mutational burden
