# Supplementary material for: An Analysis of G3BP2 in Non-Small Cell Lung Cancer
Source: Cancers (Basel). 2026 Mar 17;18(6):969. doi: 10.3390/cancers18060969 (PMC13024974; doi:10.3390/cancers18060969)
Supplement: Supplementary file 1 [file cancers-18-00969-s001.zip › Figure S1.pdf]

A

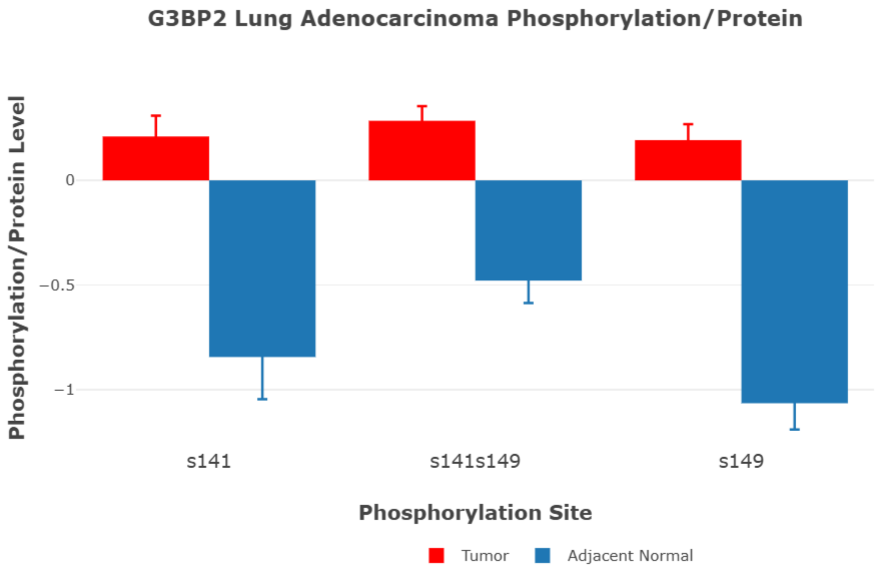
G3BP2 Lung Adenocarcinoma Phosphorylation/Protein
Phosphorylation/Protein Level
0
−0.5
−1
s141
s141s149
s149
Phosphorylation Site
Tumor
Adjacent Normal

LUAD

B

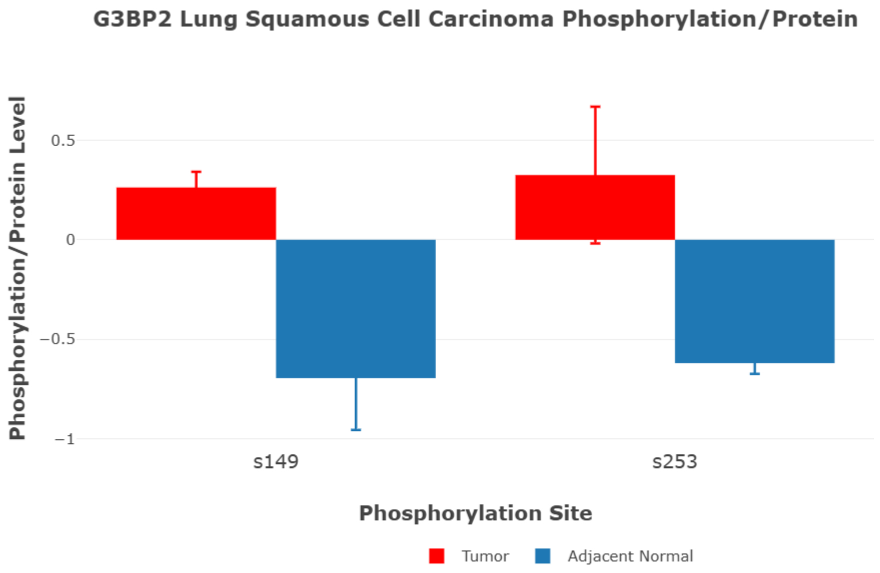
G3BP2 Lung Squamous Cell Carcinoma Phosphorylation/Protein
Phosphorylation/Protein Level
0.5
0
−0.5
−1
s149
s253
Phosphorylation Site
Tumor
Adjacent Normal

LUSC
