# Supplementary material for: An Analysis of G3BP2 in Non-Small Cell Lung Cancer
Source: Cancers (Basel). 2026 Mar 17;18(6):969. doi: 10.3390/cancers18060969 (PMC13024974; doi:10.3390/cancers18060969)
Supplement: Supplementary file 1 [file cancers-18-00969-s001.zip › Figure S3.pdf]

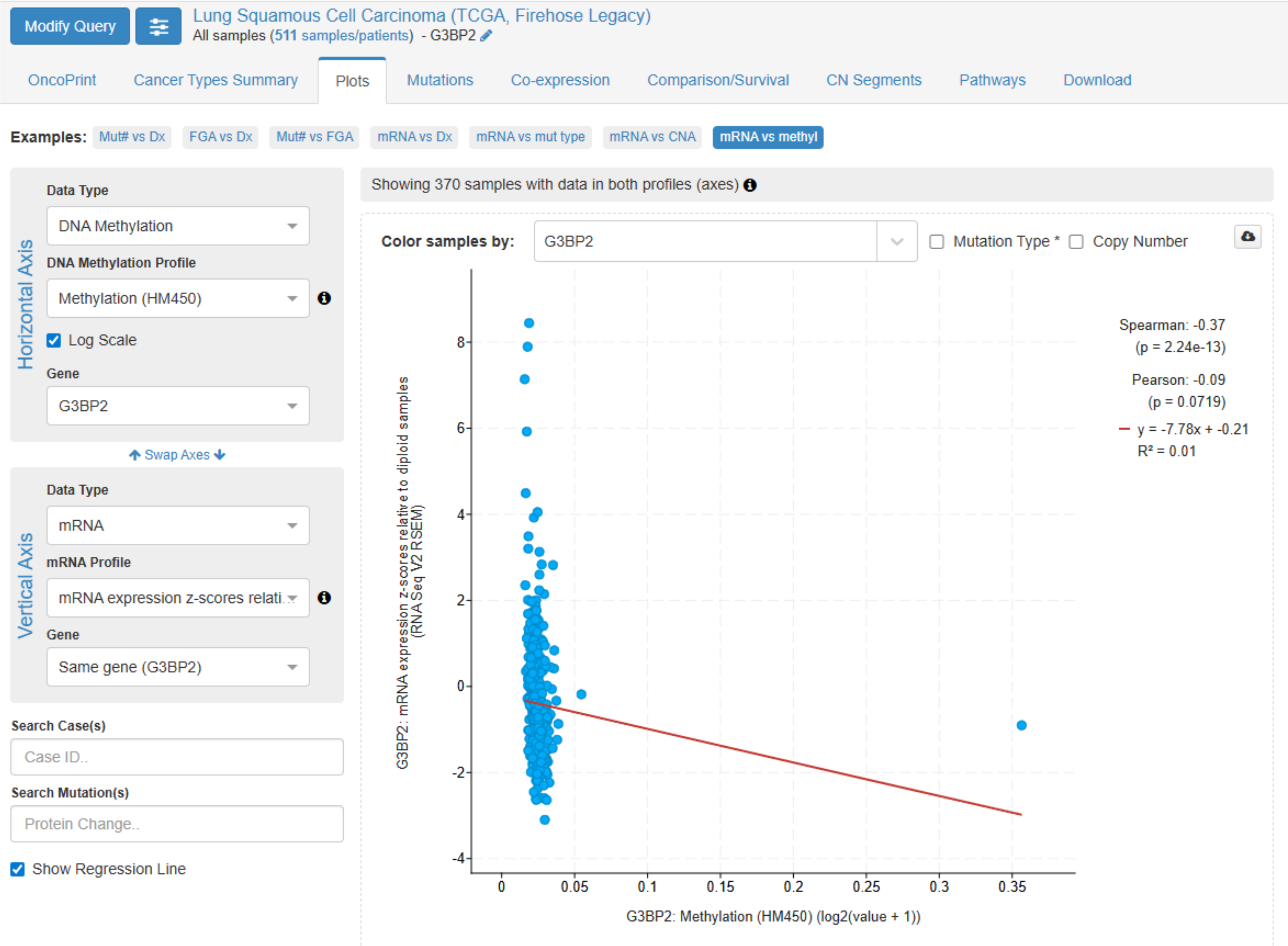

Modify Query
Lung Squamous Cell Carcinoma (TCGA, Firehose Legacy)
All samples (511 samples/patients) - G3BP2
OncoPrint
Cancer Types Summary
Plots
Mutations
Co-expression
Comparison/Survival
CN Segments
Pathways
Download
Examples:
Mut# vs Dx
FGA vs Dx
Mut# vs FGA
mRNA vs Dx
mRNA vs mut type
mRNA vs CNA
mRNA vs methyl
Horizontal Axis
Data Type
DNA Methylation
DNA Methylation Profile
Methylation (HM450)
Log Scale
Gene
G3BP2
Swap Axes
Vertical Axis
Data Type
mRNA
mRNA Profile
mRNA expression z-scores relati...
Gene
Same gene (G3BP2)
Search Case(s)
Case ID..
Search Mutation(s)
Protein Change..
Show Regression Line
Showing 370 samples with data in both profiles (axes)
Color samples by:
G3BP2
Mutation Type *
Copy Number
Spearman: -0.37
(p = 2.24e-13)
Pearson: -0.09
(p = 0.0719)
y = -7.78x + -0.21
R² = 0.01
G3BP2: mRNA expression z-scores relative to diploid samples
(RNA Seq V2 RSEM)
G3BP2: Methylation (HM450) (log2(value + 1))
