# Supplementary material for: An Analysis of G3BP2 in Non-Small Cell Lung Cancer
Source: Cancers (Basel). 2026 Mar 17;18(6):969. doi: 10.3390/cancers18060969 (PMC13024974; doi:10.3390/cancers18060969)
Supplement: Supplementary file 1 [file cancers-18-00969-s001.zip › Figure S4.pdf]

G3BP2 promoter methylation profile based on Sample types

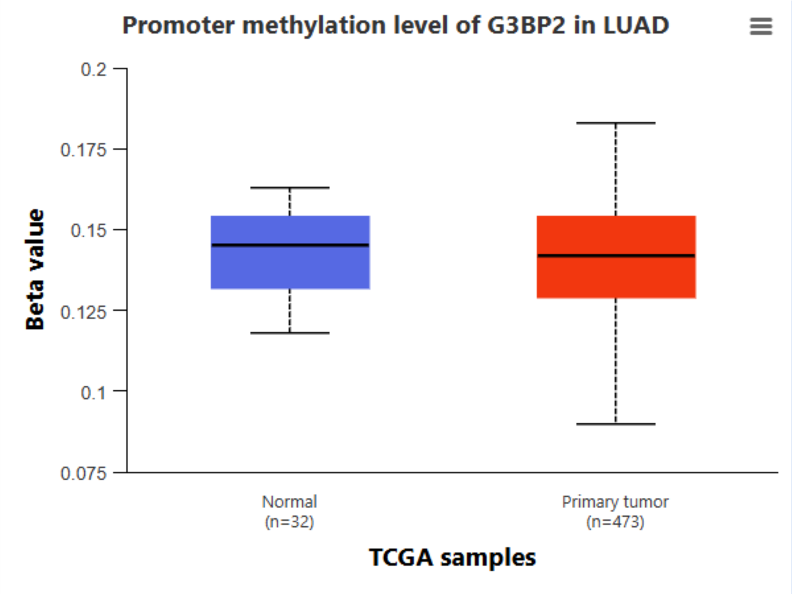

The Beta value indicates level of DNA methylation ranging from 0 (unmethylated) to 1 (fully methylated). Different beta value cut-off has been considered to indicate **hyper-methylation** [Beta value: 0.7 - 0.5] or **hypo-methylation** [Beta-value: 0.3 - 0.25]. **PMID:** 29027401, 23291739

PROBES

Illumina Id	RefGene_group
cg11489580	TSS1500,TSS200
cg01896579	TSS1500
cg06062125	TSS1500,TSS200
cg27283708	TSS200
cg00507354	TSS200,TSS1500
cg01331554	TSS200
cg00087098	TSS1500
cg21698840	TSS1500
cg04301682	TSS200,TSS1500
cg24430528	TSS1500
cg23098131	TSS1500,TSS200
cg03093398	TSS200
cg21089669	TSS1500

Comparison	Statistical significance
Normal-vs-Primary	8.726400E-02
