# Supplementary material for: An Analysis of G3BP2 in Non-Small Cell Lung Cancer
Source: Cancers (Basel). 2026 Mar 17;18(6):969. doi: 10.3390/cancers18060969 (PMC13024974; doi:10.3390/cancers18060969)
Supplement: Supplementary file 1 [file cancers-18-00969-s001.zip › Figure S7.pdf]

Image ID: 0003674_03
Acquire Time: 08-Jan-2026 12:50:28

Acquisition Information

#	Image ID	Acquire Time	Channels	Integration Times	Analysis	Image Name	Comment
1	0003674_03	08-Jan-2026 12:50:28	600	00:30	Manual	0003674_03	G3BP2 in NSCLC cell line panel

Image Display Values

Channel	Color	Minimum	Maximum	K
600	Gray Scale (Black on White)	0.0215	0.205	0

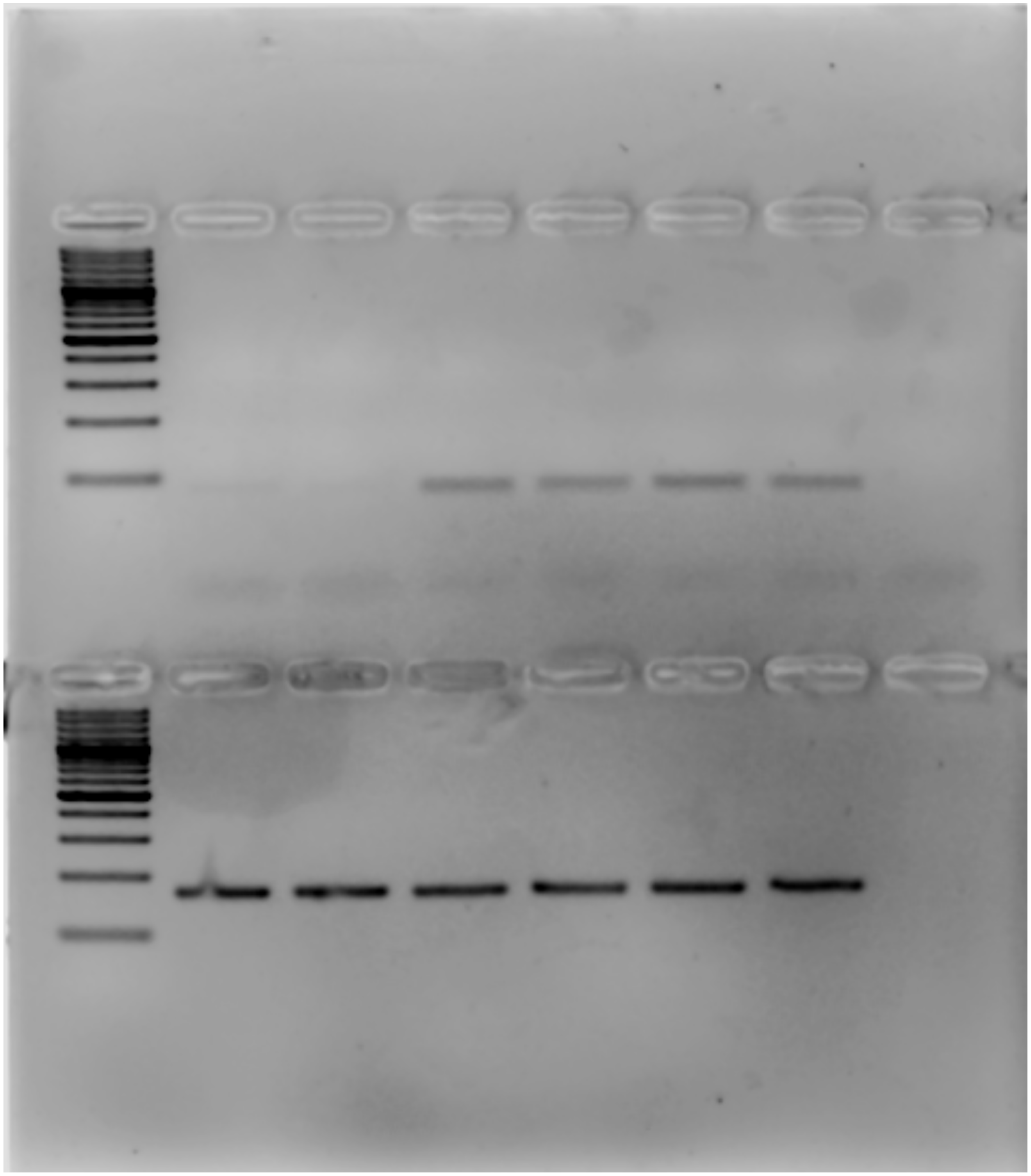

Acquisition Information (continued)

#	Image Modifications
1	Free Rotate 359 Image ID: 0003674_01; Crop Image ID: 0003674_02
