# Supplementary material for: An Analysis of G3BP2 in Non-Small Cell Lung Cancer
Source: Cancers (Basel). 2026 Mar 17;18(6):969. doi: 10.3390/cancers18060969 (PMC13024974; doi:10.3390/cancers18060969)
Supplement: Supplementary file 1 [file cancers-18-00969-s001.zip › Supplementary Figure Legends.pdf]

Supplementary Figure Legends.

Supplementary Figure S1. Phosphoproteomic analysis of G3BP2.
Phosphorylation site analysis of G3BP2 in (A) LUAD; and (B) LUSC on CPTAC proteomic data. The analysis was conducted on cProSite.

Supplementary Figure S2. DNA methylation correlations with G3BP2 mRNA expression in LUAD
Correlations between G3BP2 mRNA and DNA methylation in the TCGA-LUAD (Firehose Legacy) dataset were examined using cBioPortal.

Supplementary Figure S3. DNA methylation correlations with G3BP2 mRNA expression in LUSC
Correlations between G3BP2 mRNA and DNA methylation in the TCGA-LUSC (Firehose Legacy) dataset were examined using cBioPortal.

Supplementary Figure S4. G3BP2 Promoter methylation analysis in LUAD
The G3BP2 promoter was assessed for altered DNA CpG methylation in the TCGA-LUAD dataset using UALCAN.

Supplementary Figure S5. G3BP2 Promoter methylation analysis in LUSC
The G3BP2 promoter was assessed for altered DNA CpG methylation in the TCGA-LUSC dataset using UALCAN.

Supplementary Figure S6. G3BP2 mRNA expression in a panel of isogenic cisplatin sensitive/resistant NSCLC cell lines.
G3BP2 mRNA was examined in a panel of cisplatin sensitive/resistant cell line using qPCR for (A) A549; (B) SKMES1 and (C) DLKP.

Supplementary Figure S7. Expression of G3BP2 in a panel of normal lung or lung cancer cell lines.
Full (uncropped) image used to generate Figure 11A.
