# Supplementary figures and images for: An Analysis of G3BP2 in Non-Small Cell Lung Cancer
Source: Cancers (Basel). 2026 Mar 17;18(6):969. doi: 10.3390/cancers18060969 (PMC13024974; doi:10.3390/cancers18060969)

**A**

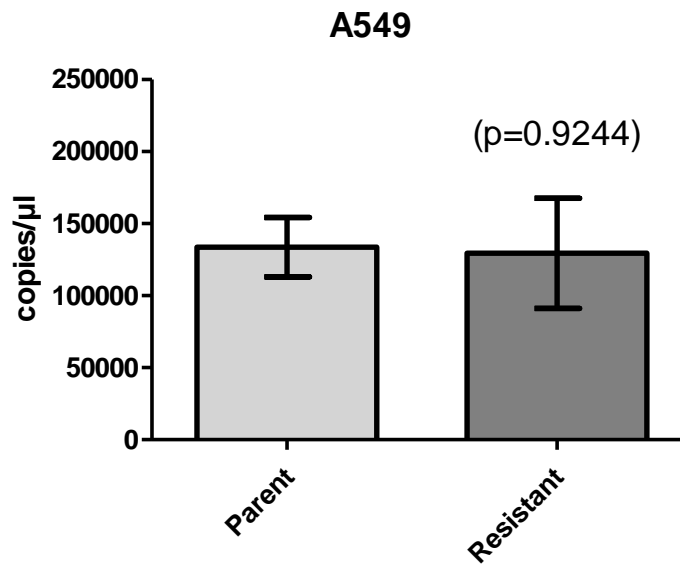

**B**

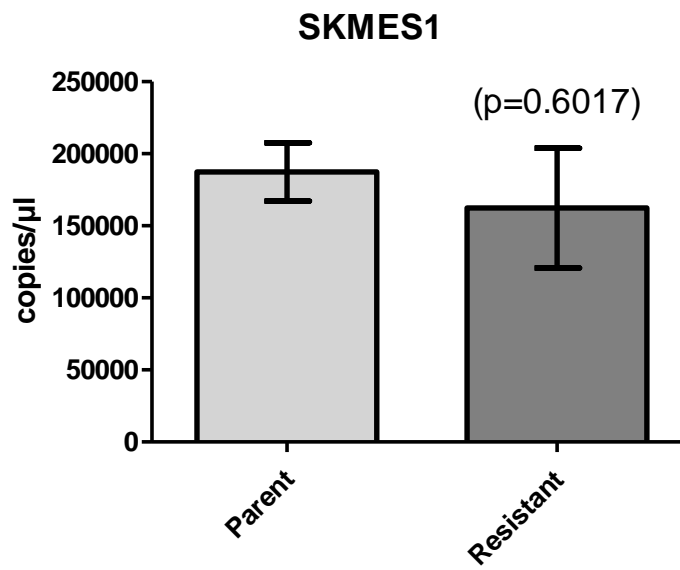

**C**

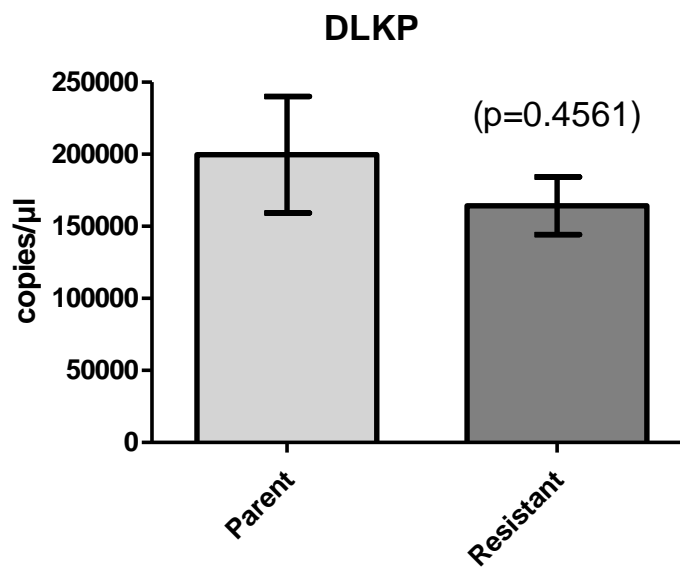

Supplement: Supplementary file 1 [file cancers-18-00969-s001.zip › Figure S6.pdf]
